# Supplementary material for: Selective neuronal degeneration in MATR3 S85C knock-in mouse model of early-stage ALS
Source: Nat Commun. 2020 Oct 20;11:5304. doi: 10.1038/s41467-020-18949-w (PMC7576598; doi:10.1038/s41467-020-18949-w)
Supplement: Supplementary file 1 — Supplementary Information [file 41467_2020_18949_MOESM1_ESM.pdf]

Supplementary Information

Selective neuronal degeneration in MATR3 S85C knock-in mouse model of early-stage ALS

Ching Serena Kao, Rebekah van Bruggen, Jihye Rachel Kim, Xiao Xiao Lily Chen, Cadia Chan, Jooyun Lee, Woo In Cho, Melody Zhao, Claudia Arndt, Katarina Maksimovic, Mashiat Khan, Qiumin Tan, Michael D. Wilson, Jeehye Park

Correspondence to: jeehye.park@sickkids.ca

This file includes:

- Supplementary Figures 1 – 16
- Supplementary Table 1: List of primers used in this study
- Supplementary Table 2: Animal numbers (n) and significance values for Figure 2a
- Supplementary References

Supplementary Figure 1

a **Matr3 deletion mice**

Mouse *Matr3* locus chr18qB2

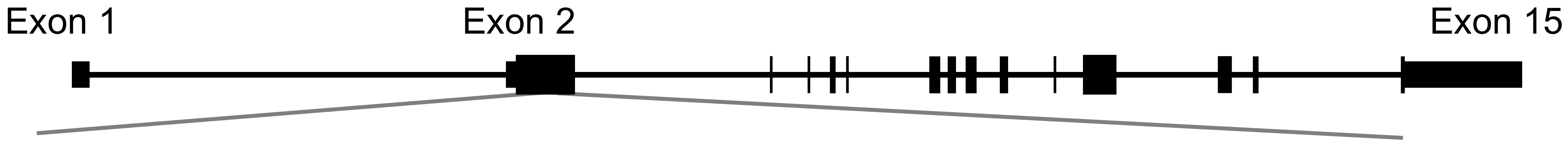

em5 p.87Lfs*34
TTTGCAGTCTATATT|TAACATT|GGAAGTAGAGGTCCAC
TTTGCAGTCTATATT|------------|GGAAGTAGAGGTCCAC

em4 p.F115Lfs*7
CCAGGCCAGTAATATTTTGGCCAG|-------|CTTTGGTCTGTCTG
CCAGGCCAGTAATATTTTGGCCAG|TCTG|CTTTGGTCTGTCTG

em3 p.L112Wfs*5
CCAGGCCAGTAATA|TTTTGGCCAGC|TTTGGTCTGTCTGC
CCAGGCCAGTAATA|-------------------|TTTGGTCTGTCTGC

b

		E18.5				P0				P>0			
		WT	HET	HOM	TOTAL	WT	HET	HOM	TOTAL	WT	HET	HOM	TOTAL
em5	Matr3pF87Lfs*34	16	20	11	47	20	39	8	67	13	12	0	25
em4	Matr3pF115Lfs*7	7	20	7	34	30	51	9	90	19	31	0	50
em3	Matr3pL112Wfs*5	24	39	18	81	33	63	13	109	7	13	0	20

c

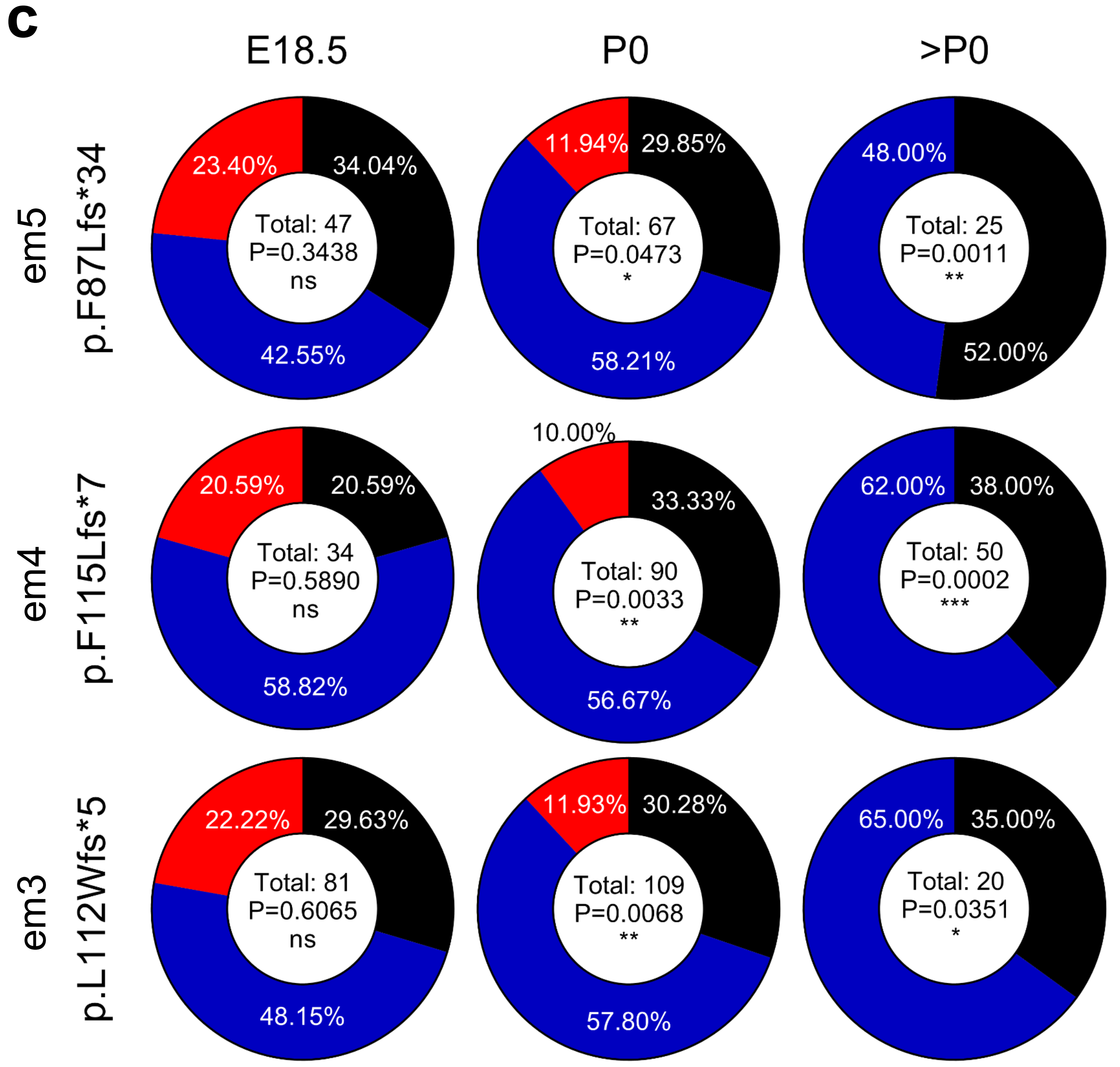

d

Homozygous MATR3 Knockout

Control

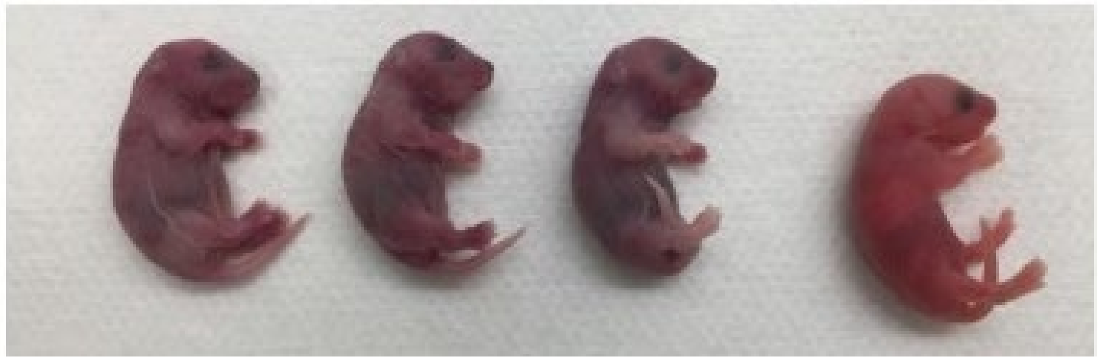

Supplementary Fig. 1 Generation of MATR3 knockout mice. **a** Using CRISPR/Cas9, three founder lines were generated by non-homologous end joining resulting in either an insertion or deletion leading to a truncated protein product. **b, c** Homozygous knockout animals for all three lines were found to be perinatal lethal, dying just after birth, resulting in skewed genotyping ratios. **c** Statistical significance was determined using the chi squared test for goodness of fit; ns = not significant, $*p< 0.05$, $**p<0.01$, $***p<0.001$. **d** Homozygous MATR3 knockout animals were cyanotic, showed labored breathing and died within a few hours after birth. Source data are provided as a Source data file.

Supplementary Figure 2

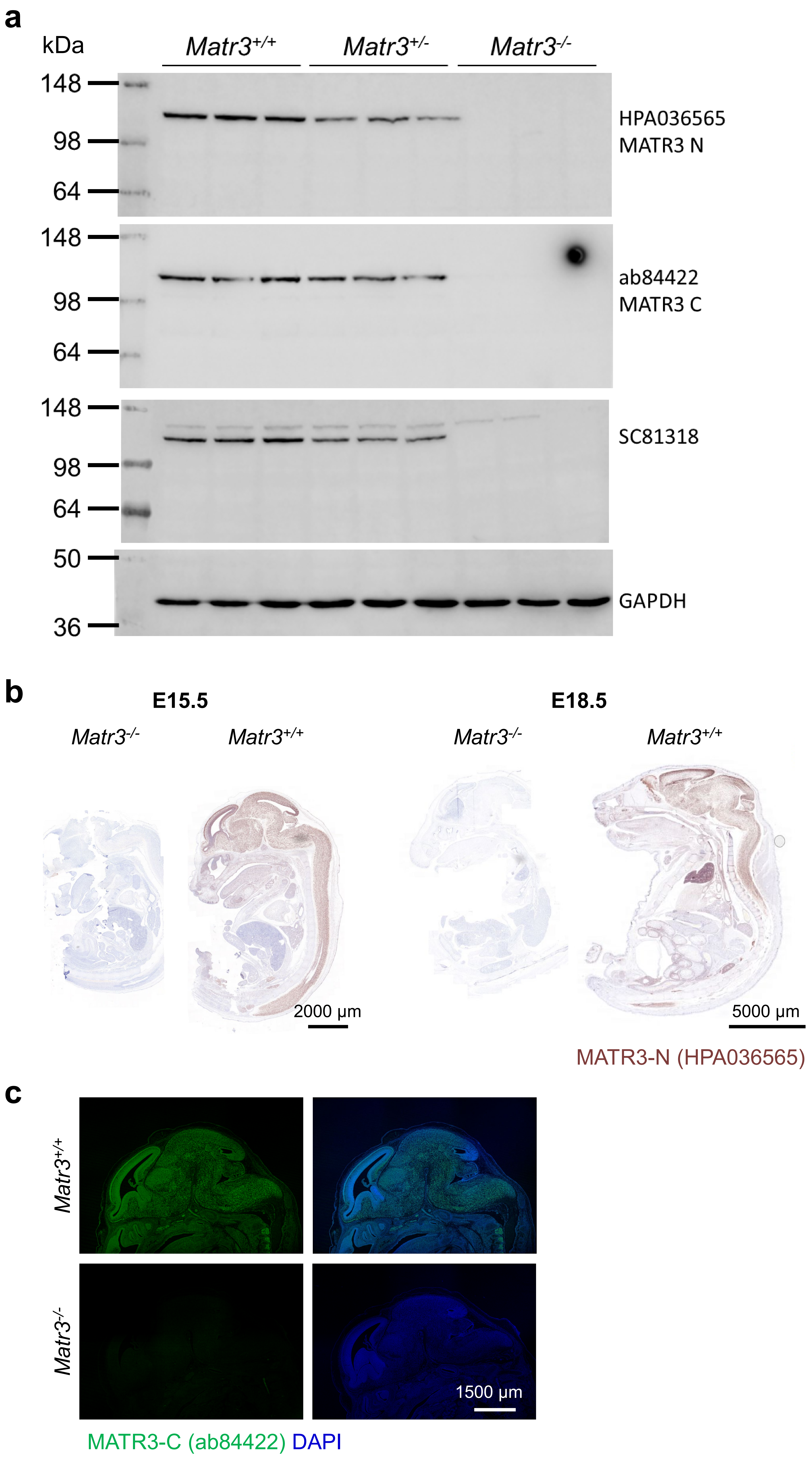

Supplementary Fig. 2 Validation of the specificity of MATR3 antibodies through western blot analysis and immunohistochemistry. **a** Western blot showing absence of MATR3 proteins in MATR3 knockout mice. Total cell lysates from three E12.5 *Matr3*$^{+/+}$, *Matr3*$^{+/-}$ and *Matr3*$^{-/-}$ embryos were probed with three different MATR3 antibodies. HPA036565 (Sigma anti-MATR3-N antibody, 1:1000), ab84422 (Abcam anti-MATR3-C antibody, 1:1000), SC81318 (Santa Cruz anti-MATR3 antibody, 1:1000) and GAPDH (Millipore, mouse monoclonal, 1:10,000) was used as a loading control. **b** Validation of the specificity of anti-MATR3-N antibody through immunohistochemistry using E15.5 and E18.5 wildtype and MATR3 knockout embryos (HPA036565, 1:200). **c** Validation of anti-MATR3-C antibody in E15.5 wildtype and MATR3 knockout brains by immunofluorescence staining (ab84422, 1:400). Source data are provided as a Source data file.

Supplementary Figure 3

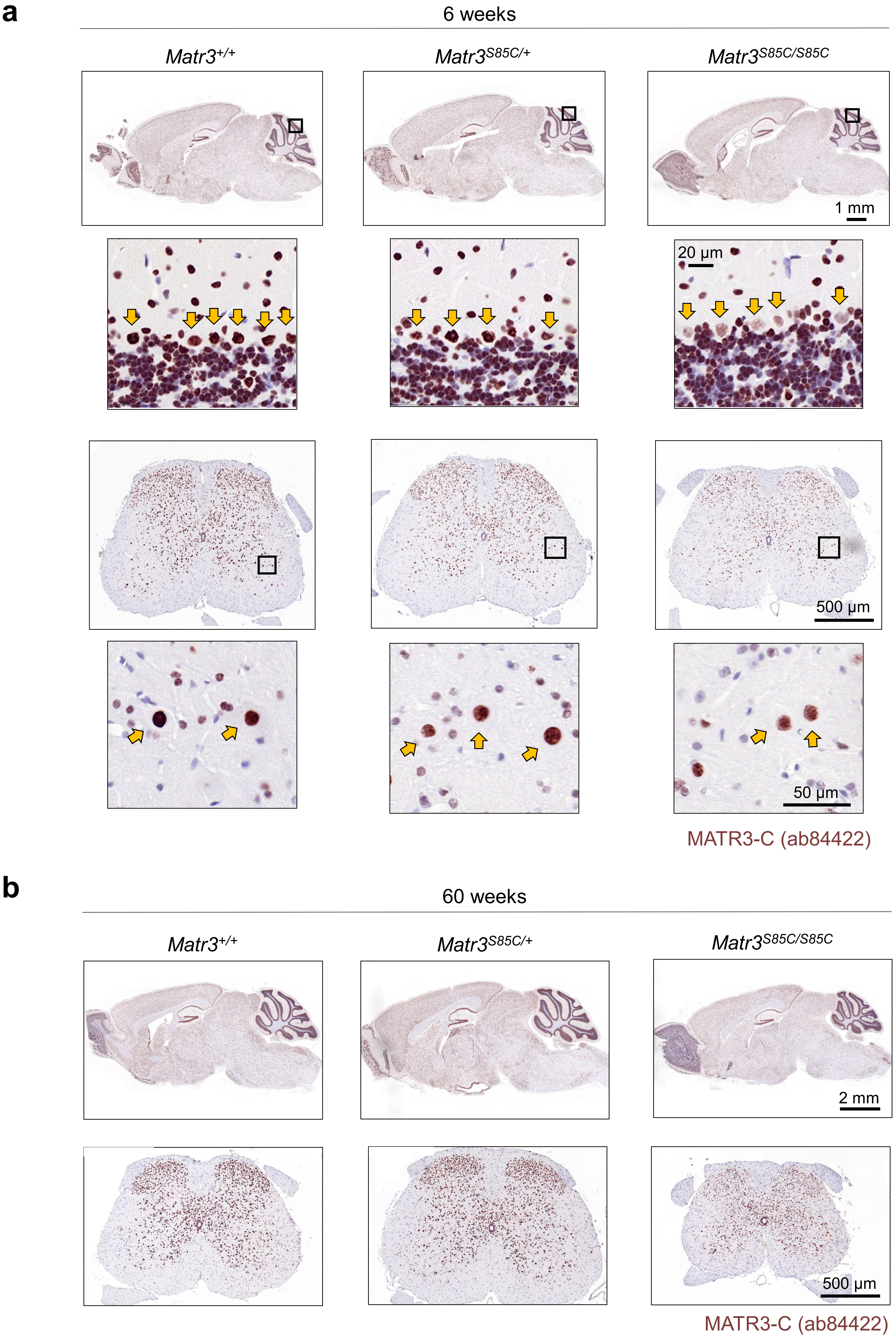

Supplementary Fig. 3 MATR3 localization and levels in the cerebellum and spinal cord of homozygous S85C mice at both 6 weeks and end-point. **a** 6 weeks old brains and lumbar spinal cords stained with anti-MATR3-C antibody. Higher magnification images show Purkinje cells or motor neurons indicated by the yellow arrows. **b** 60 weeks old brains and lumbar spinal cords stained with anti-MATR3-C antibody.

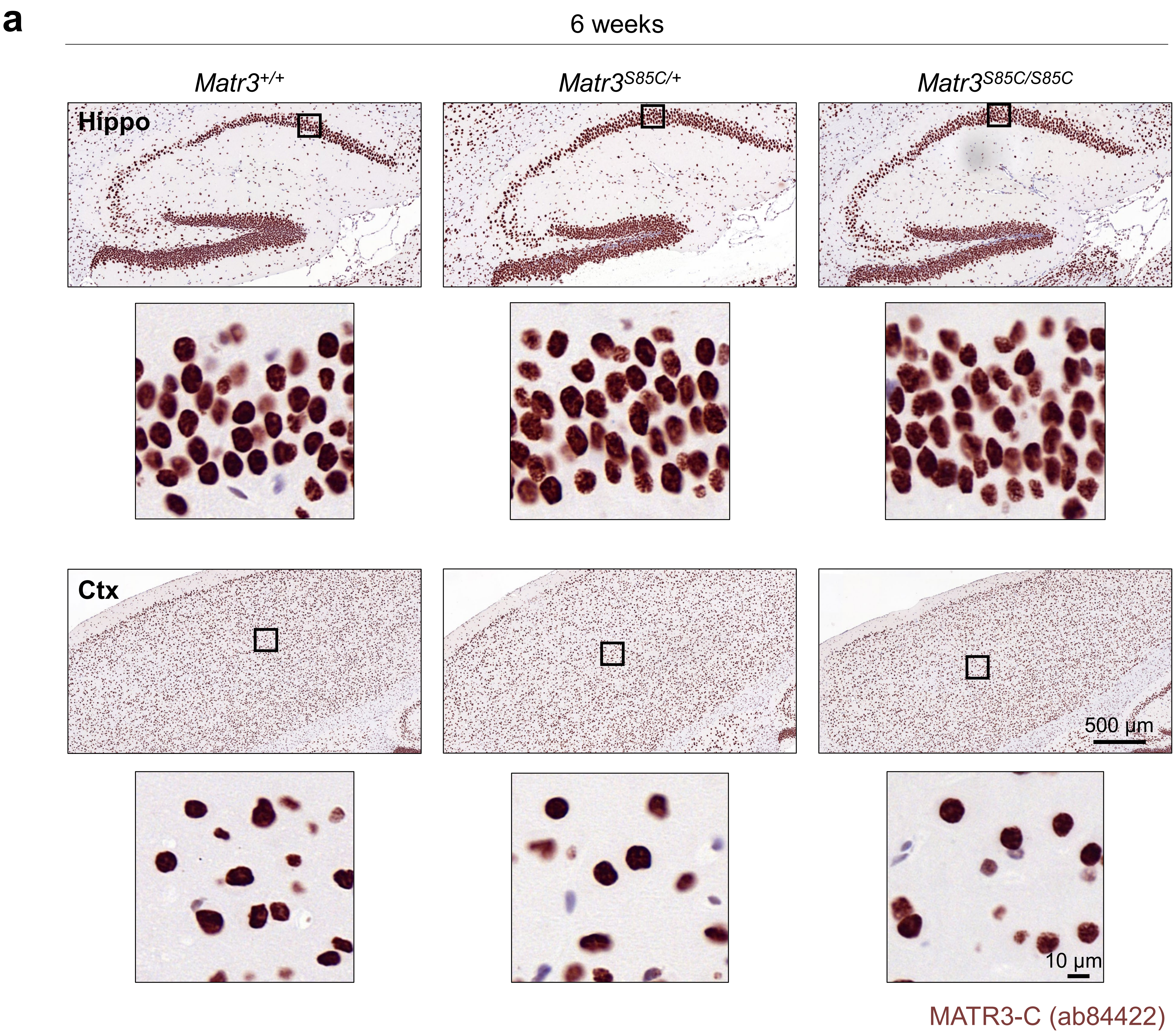

Supplementary Fig. 4 MATR3 is expressed in the hippocampus (hippo) and cortex (ctx) of wildtype, heterozygous and homozygous S85C mice. **a** 6 weeks old hippocampus (hippo) and cortex (ctx) stained with anti-MATR3-C antibody. Higher magnification images show CA1 neurons in the hippocampus and neurons in the prefrontal cortex.

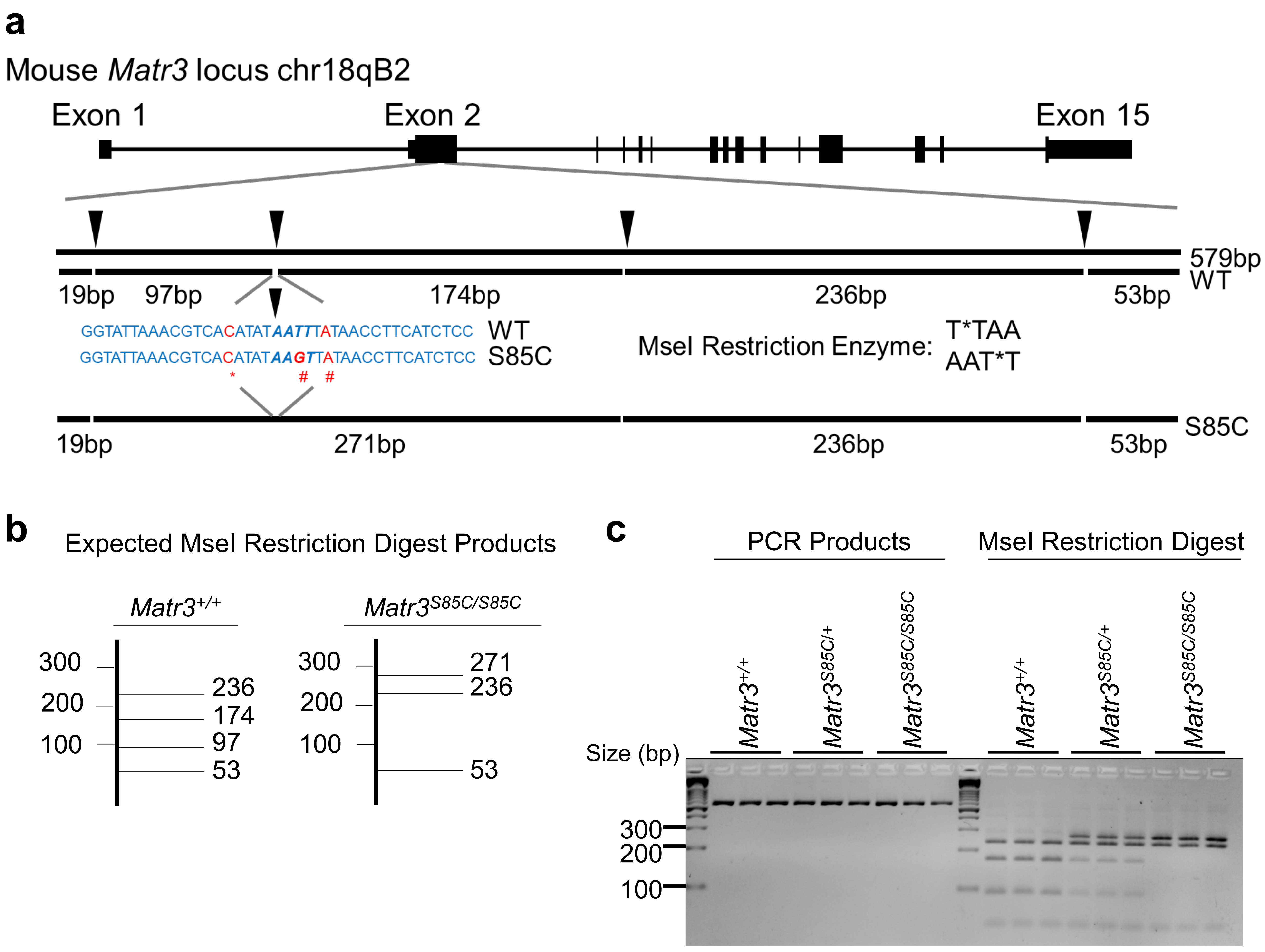

Supplementary Fig. 5 Genotyping the S85C allele. **a** Schematic representation of the MseI Restriction enzyme genotyping. A 579 bp region of DNA was PCR amplified prior to digestion with MseI restriction enzyme (cut site: T*TAA/AAT*T). **b** The expected MseI restriction digest results for wildtype (*Matr3*$^{+/+}$) and S85C homozygous (*Matr3*$^{S85C/S85C}$). **c** PCR product and MseI restriction digest results. Source data are provided as a Source data file.

Supplementary Figure 6

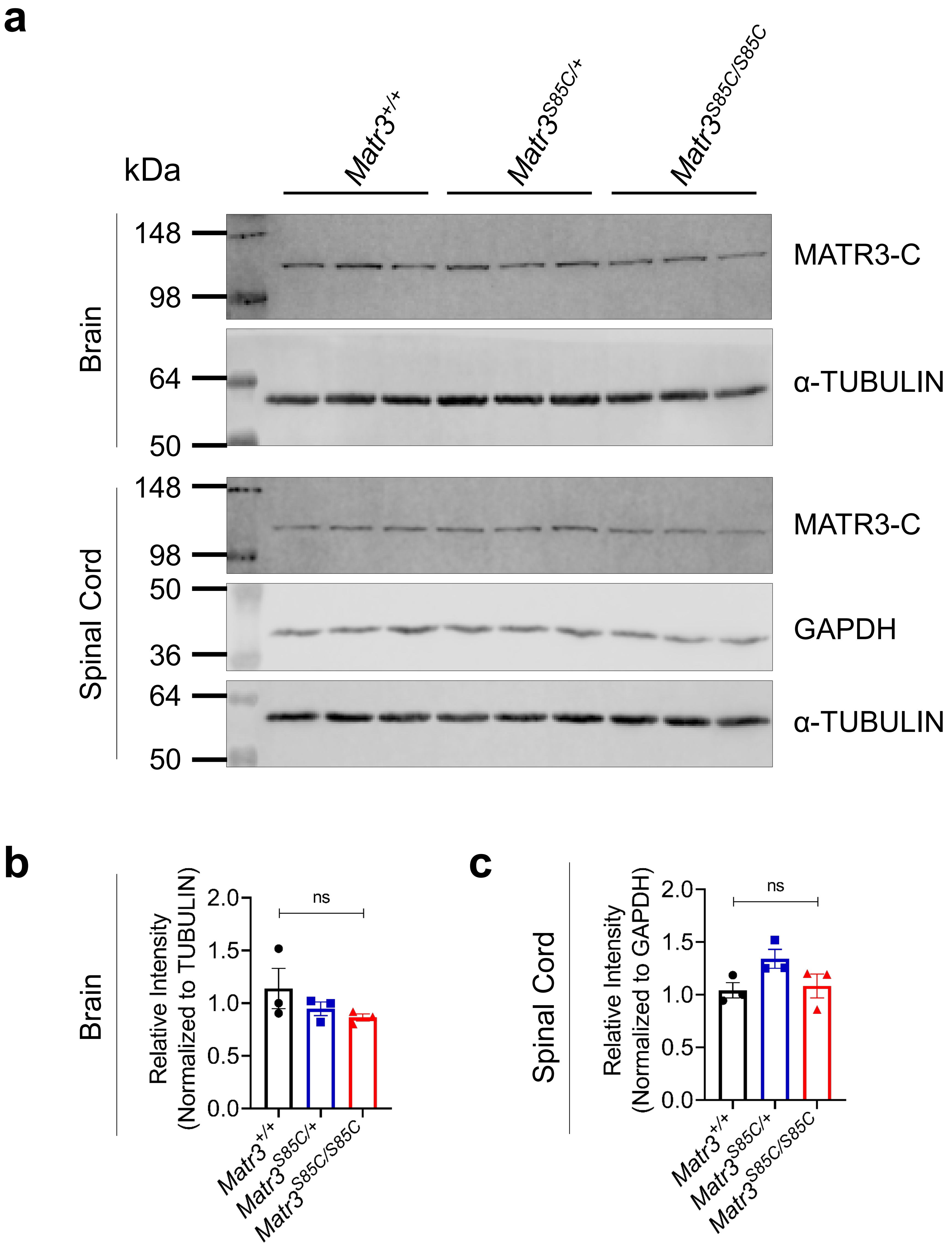

Supplementary Fig. 6 MATR3 protein levels are not significantly different in the brain and spinal cord of 3 weeks old littermates. **a** Western blot showing MATR3 protein levels with anti-MATR3-C antibody (ab84422) and either α-tubulin or GAPDH antibodies as a loading control. **b**, **c** Quantification of MATR3 protein levels at 3 weeks of age in the (**b)** brain compared to α-tubulin or (**c**) spinal cord, compared to GAPDH. Data presented as mean ± SEM (n = 3 biological replicates per genotype). Statistical significance was determined using the unpaired two-tailed *t*-test; ns = not significant. Source data are provided as a Source data file.

Supplementary Figure 7

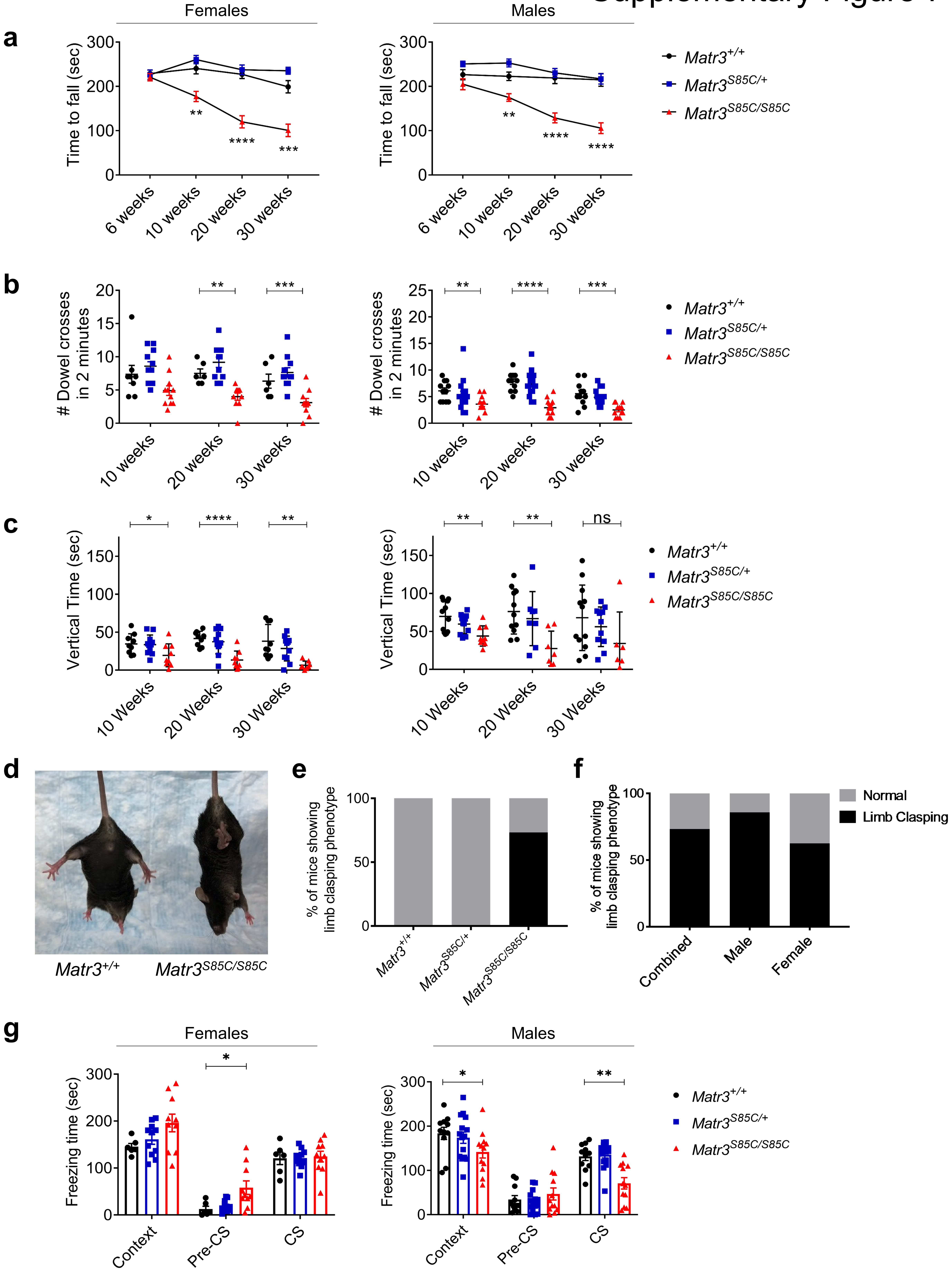

Supplementary Fig. 7 Female and male homozygous S85C mice exhibit motor deficits. **a, b** Homozygous S85C knock-in mice exhibit progressive motor deficits starting as early as 10 weeks of age for both female and male mice as measured by (**a**) Rotarod (males: 6, 10, 20, and 30 weeks of age: n=11 *Matr3*$^{+/+}$, 16 *Matr3*$^{S85C/+}$, 12 *Matr3*$^{S85C/S85C}$, $^{**}p$=0.0012, $^{****}p$<0.0001; females: 6 weeks: n=9 *Matr3*$^{+/+}$, 14 *Matr3*$^{S85C/+}$, 12 *Matr3*$^{S85C/S85C}$, 10 weeks: n=8 *Matr3*$^{+/+}$, 13 *Matr3*$^{S85C/+}$, 11 *Matr3*$^{S85C/S85C}$, $^{**}p$=0.0017, 20 and 30 weeks: n=6 *Matr3*$^{+/+}$, 11 *Matr3*$^{S85C/+}$, 10 *Matr3*$^{S85C/S85C}$, $^{****}p$<0.0001, $^{***}p$=0.0004) and (**b)** dowel test (males: n=10, 20 and 30 weeks of age: n=11 *Matr3*$^{+/+}$, 16 *Matr3*$^{S85C/+}$, 12 *Matr3*$^{S85C/S85C}$, $^{**}p$=0.0019, $^{****}p$<0.0001, $^{***}p$=0.0004; females: 10 weeks: n=8 *Matr3*$^{+/+}$, 12 *Matr3*$^{S85C/+}$, 11 *Matr3*$^{S85C/S85C}$, 20 weeks: n=6 *Matr3*$^{+/+}$, 11 *Matr3*$^{S85C/+}$, 10 *Matr3*$^{S85C/S85C}$, $^{**}p$=0.0012, 30 weeks: n=6 *Matr3*$^{+/+}$, 11 *Matr3*$^{S85C/+}$, 10 *Matr3*$^{S85C/S85C}$, $^{***}p$=0.0132). **c** Rearing time (time spent standing on hind-limbs) at 10, 20 and 30 weeks of age, was measured using OpenField analysis (males: 10 weeks: n=12 *Matr3*$^{+/+}$, 11 *Matr3*$^{S85C/+}$, 9 *Matr3*$^{S85C/S85C}$, $^{**}p$=0.0035, 20 weeks: n=11 *Matr3*$^{+/+}$, 8 *Matr3*$^{S85C/+}$, 7 *Matr3*$^{S85C/S85C}$, $^{**}p$=0.0020, 30 weeks: n=12 *Matr3*$^{+/+}$, 11 *Matr3*$^{S85C/+}$, 6 *Matr3*$^{S85C/S85C.}$; females: 10 and 20 weeks: n=9 *Matr3*$^{+/+}$, 11 *Matr3*$^{S85C/+}$, 9 *Matr3*$^{S85C/S85C}$, $^{*}p$=0.0403, $^{****}p$<0.0001, 30 weeks: n=9 *Matr3*$^{+/+}$, 11 *Matr3*$^{S85C/+}$, 8 *Matr3*$^{S85C/S85C}$, $^{**}p$=0.0013) . **d** Image showing hind-limb clasping phenotype in 20 week old homozygous S85C mouse. **e** Graph shows percentage of 20 week old mice exhibiting hind-limb clasping (n=24 *Matr3*$^{+/+}$, 19 *Matr3*$^{S85C/+}$, 15 *Matr3*$^{S85C/S85C}$). **f** Graph shows percentage of 20 week old male and female homozygous S85C mice exhibiting hind-limb clasping (males: n=7; females: n=8). **g** Aversive memory was measured for 40 week old male and female animals by measuring the freezing time in either the same context (context) or in a different context before (Pre-CS) or after (CS) the conditional stimulus (males: n=11 *Matr3*$^{+/+}$, 15 *Matr3*$^{S85C/+}$, 12 *Matr3*$^{S85C/S85C}$, $^{*}p$=0.0418, $^{**}p$=0.0011; females: n=6 *Matr3*$^{+/+}$, 11 *Matr3*$^{S85C/+}$, 10 *Matr3*$^{S85C/S85C}$, $^{*}p$=0.0342). (**a**) Data is represented as mean ± s.e.m, with each dot (**b, c, g**) representing a single animal. Significance was determined by unpaired two-tailed *t*-test. Source data are provided as a Source data file.

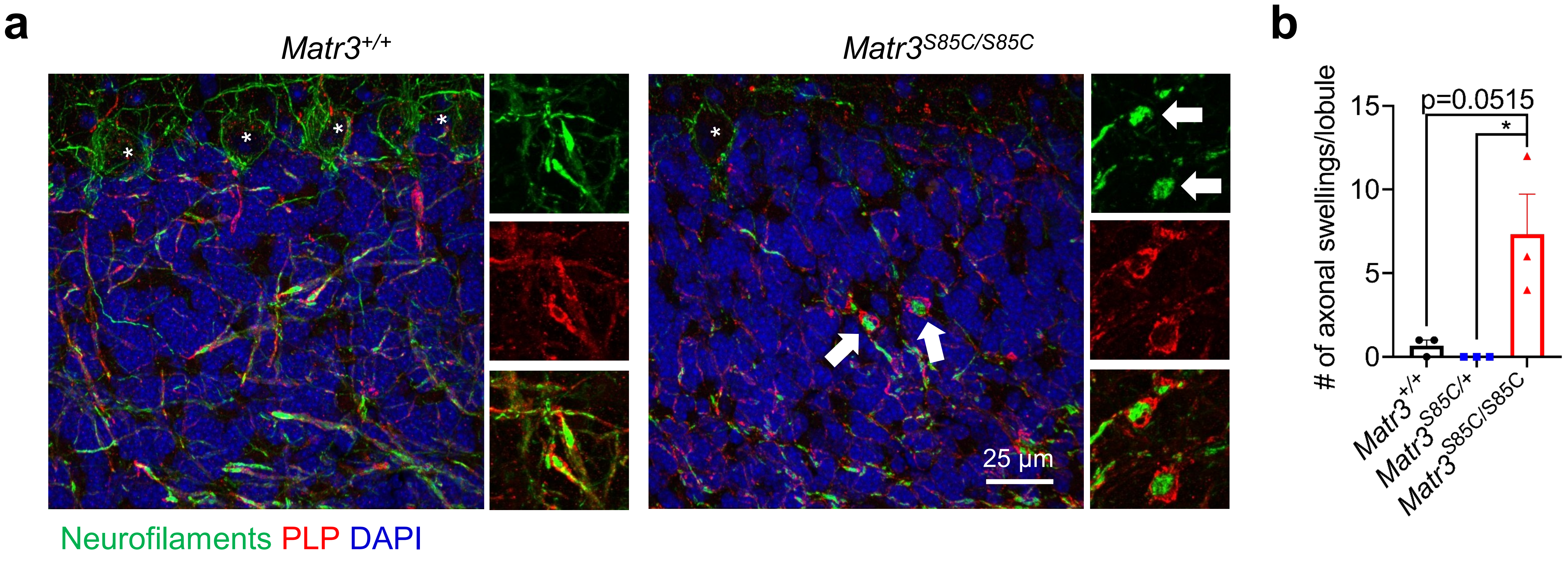

Supplementary Fig. 8 Axonal spheroids were observed in the granular layer of the cerebellum in homozygous S85C mice. **a** Images showing Purkinje cells (white asterisks) and axonal swellings ensheathed with myelin (white arrows) in the granular layer. Insets show higher magnification images of myelinated axons in granular layer. **b** Graph showing the number of axonal swellings in the lobule (n=3 *Matr3*$^{+/+}$, 3 *Matr3*$^{S85C/+}$, 3 *Matr3*$^{S85C/S85C}$, *p = 0.038). Data is represented as mean ± s.e.m. and each dot represents a single mouse. Significance was determined by unpaired two-tailed *t*-test. Source data are provided as a Source data file.

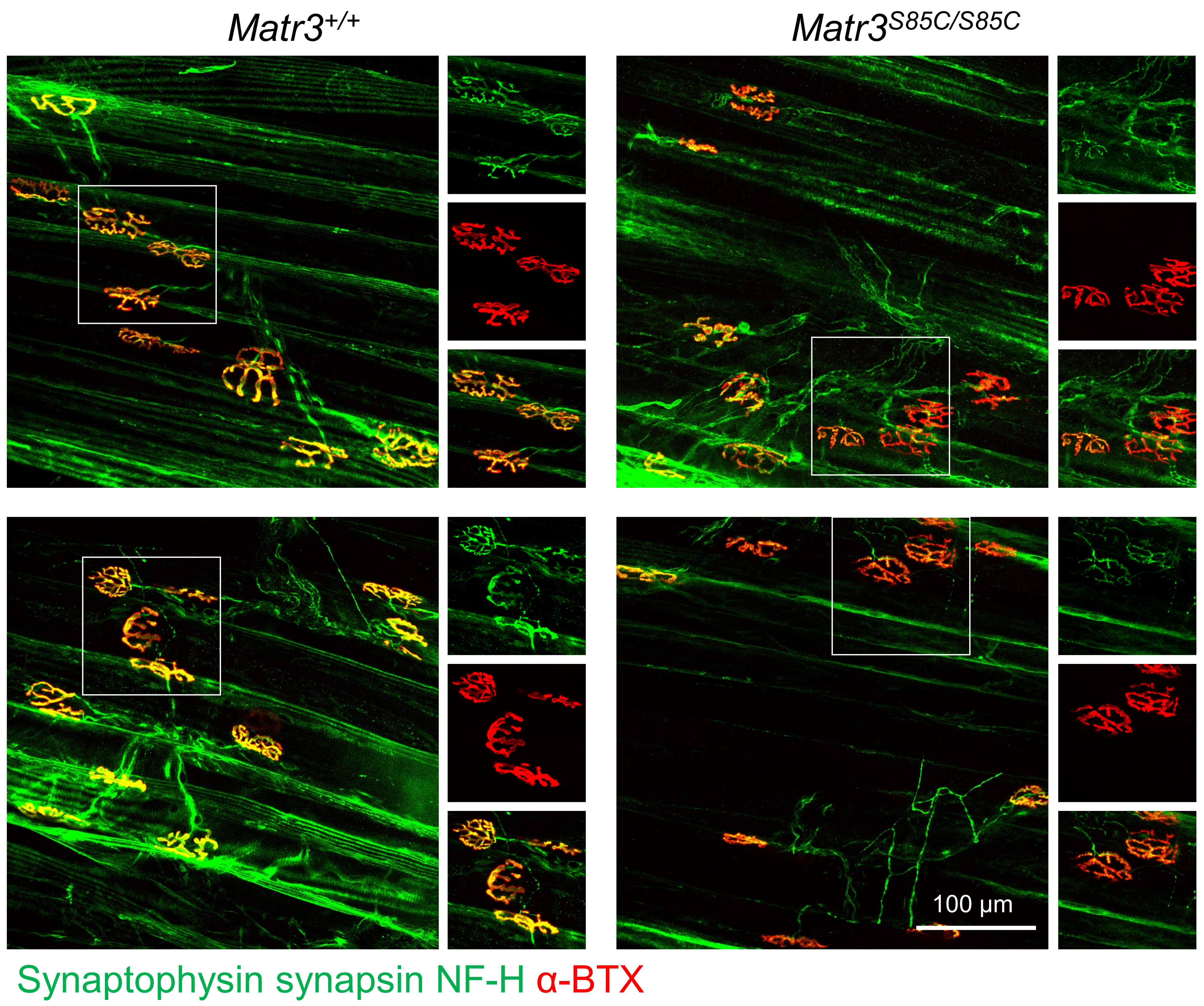

Supplementary Fig. 9 Partial denervation of the motor neurons in the tibialis anterior (TA) muscle in *Matr3*$^{S85C/S85C}$ mice. Representative images showing synaptophysin and synapsin staining of pre-synaptic terminals, neurofilament H staining of presynaptic axons and α-bungarotoxin staining of post-synaptic terminals in the TA muscles at end-stage. Magnified images show innervated endplate in *Matr3*$^{+/+}$ and partially denervated endplate in *Matr3*$^{S85C/S85C}$ mice.

Supplementary Figure 10

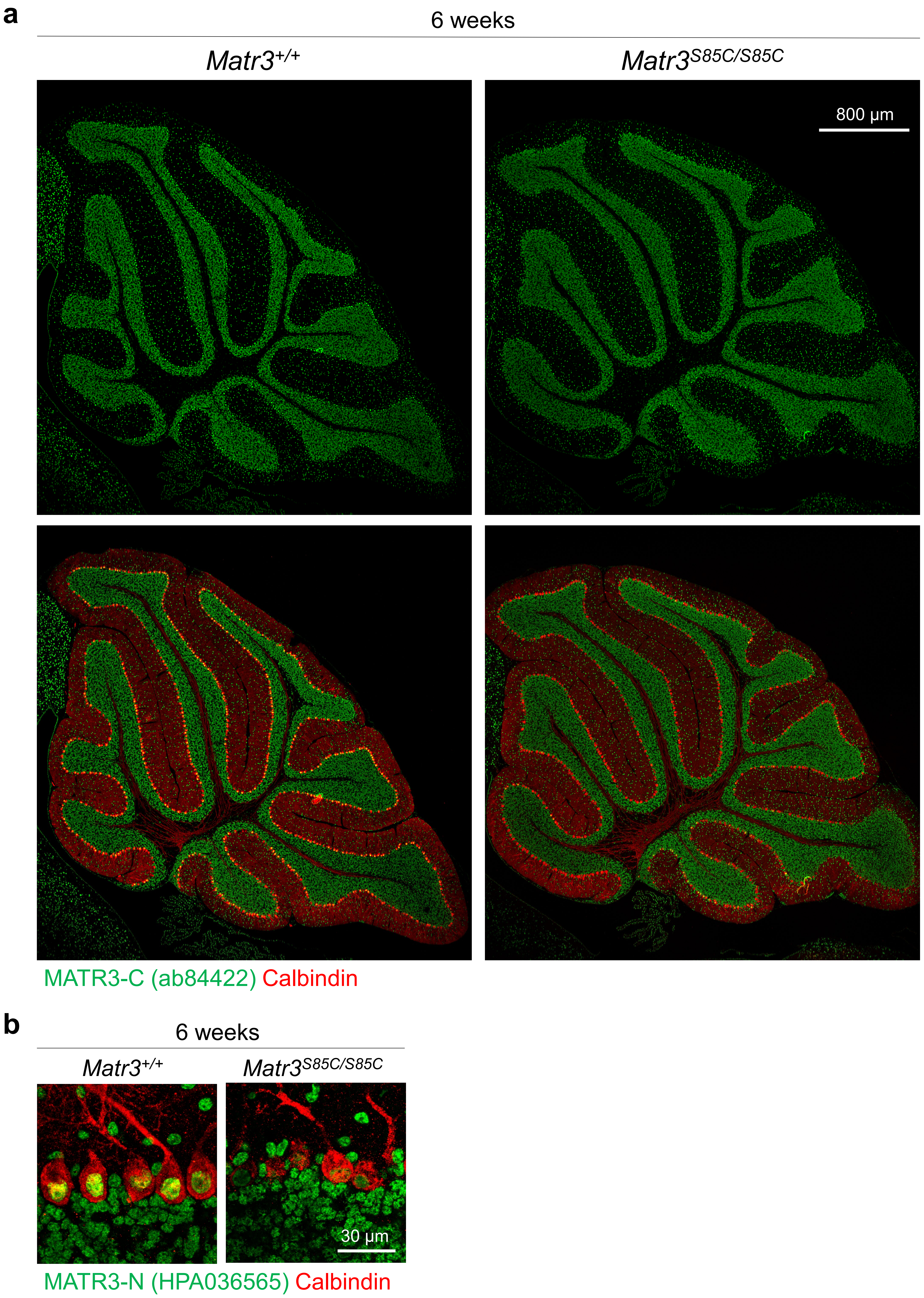

Supplementary Fig. 10 MATR3 staining in Purkinje cells of the cerebellum of homozygous S85C mice is reduced at 6 weeks of age. **a** Representative images showing MATR3 staining (detected with MATR3-C antibody) throughout the cerebellum of 6-week-old mice, including calbindin-positive Purkinje cells. **b** Representative images showing MATR3 staining (detected with MATR3-N antibody) in the Purkinje cells of 6 weeks old mice.

Supplementary Figure 11

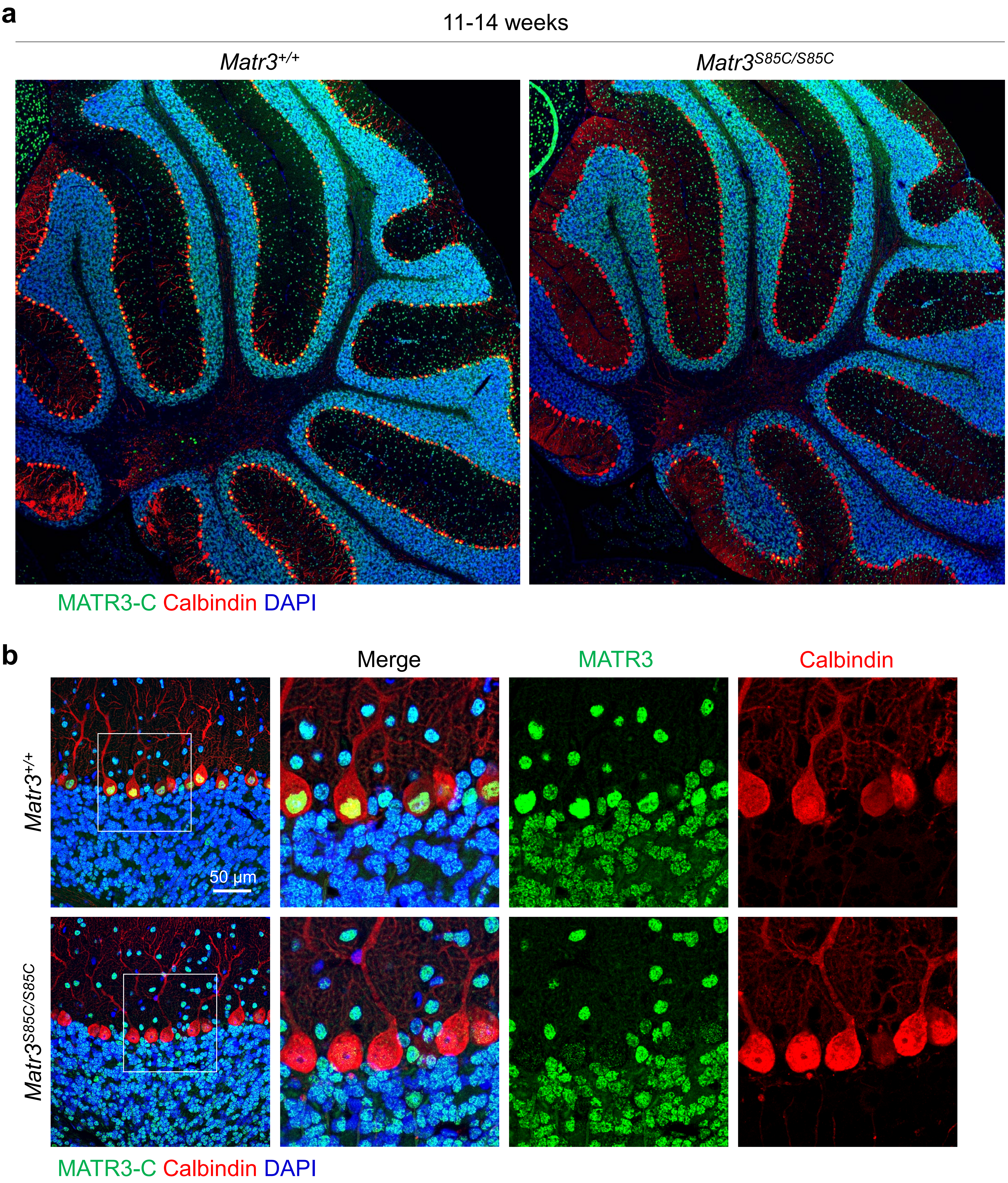

Supplementary Fig. 11 MATR3 staining in the cerebellum of 11 to 14-week-old mice. **a** Representative images showing MATR3 staining (detected with MATR3-C antibody) throughout the cerebellum. **b** Representative images showing MATR3 staining (detected with MATR3-C antibody) in the Purkinje cells (marked by calbindin) and other cells in the cerebellum.

Supplementary Figure 12

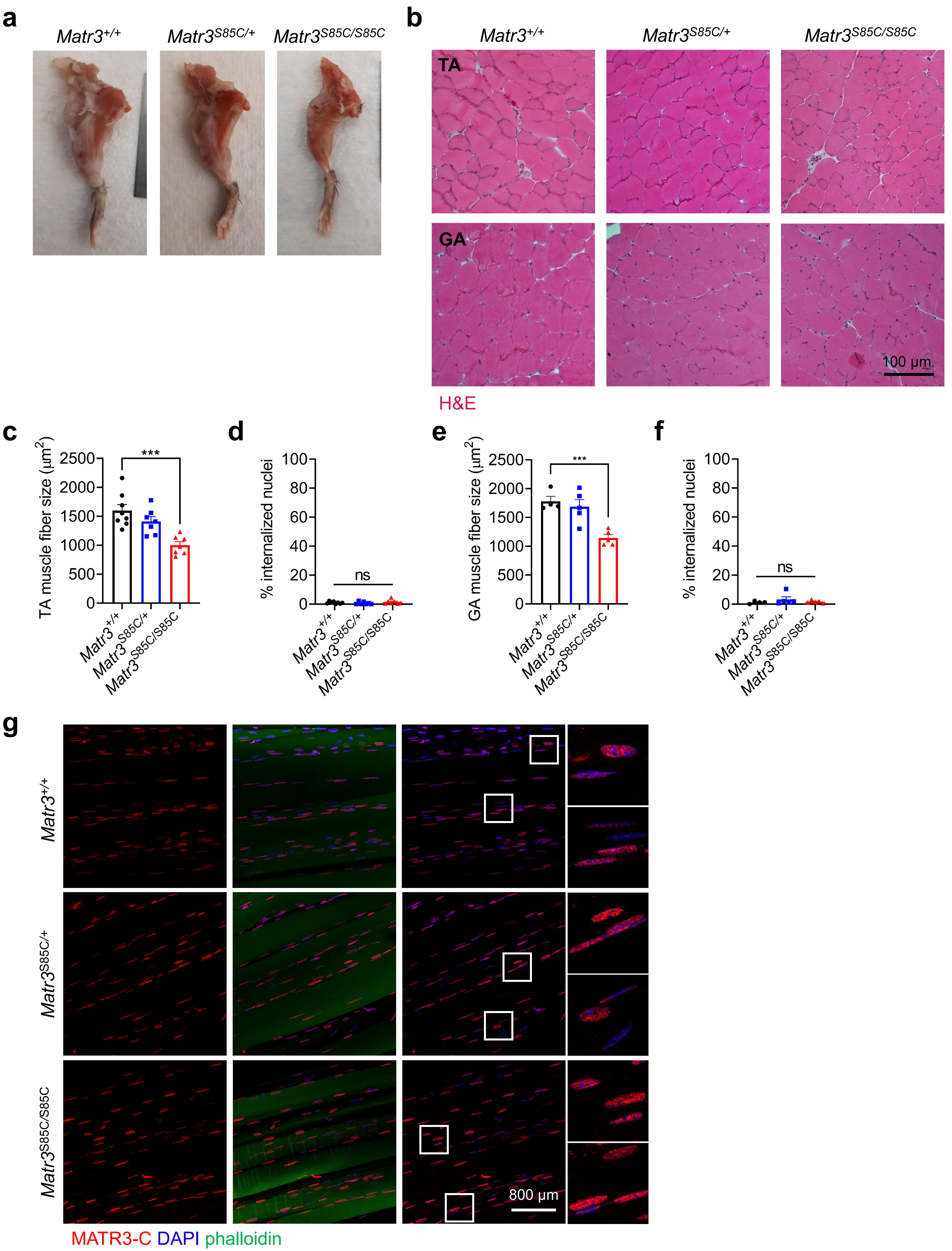

Supplementary Fig. 12 Muscle atrophy without prominent muscle pathology in end-stage *Matr3*$^{S85C/S85C}$ animals. **a** Hind-limb size differences, showing muscle atrophy in *Matr3*$^{S85C/S85C}$ mice. **b** Representative images of hematoxylin and eosin stained TA and gastrocnemius (GA) muscles. **c, d** Graphs show (**c**) average muscle fiber size and (**d)** percentage of internalized nuclei in the TA muscles (n= 8 *Matr3*$^{+/+}$, 7 *Matr3*$^{S85C/+}$, 7 *Matr3*$^{S85C/S85C}$, ***p = 0.0003, ns = not significant*).* Data presented as mean ± s.e.m. and each dot represents a single animal. Significant was determined by unpaired two-tailed *t*-test. **e, f** Graphs show (**e**) average muscle fiber size and (**f)** percentage of internalized nuclei in the gastrocnemius (GA) muscles (n= 4 *Matr3*$^{+/+}$, 5 *Matr3*$^{S85C/+}$, 5 *Matr3*$^{S85C/S85C}$,***p = 0.0004, ns = not significant). Data presented as mean ± s.e.m and each dot represents a single animal. Significance determined by unpaired two-tailed *t*-test. **g** Representative images of MATR3 localization in TA muscle. MATR3 shown in red (detected with MATR3-C antibody), DAPI in blue, and phalloidin in green.

Supplementary Figure 13

a Cerebellum *Matr3*$^{S85C/+}$ vs *Matr3*$^{S85C/S85C}$

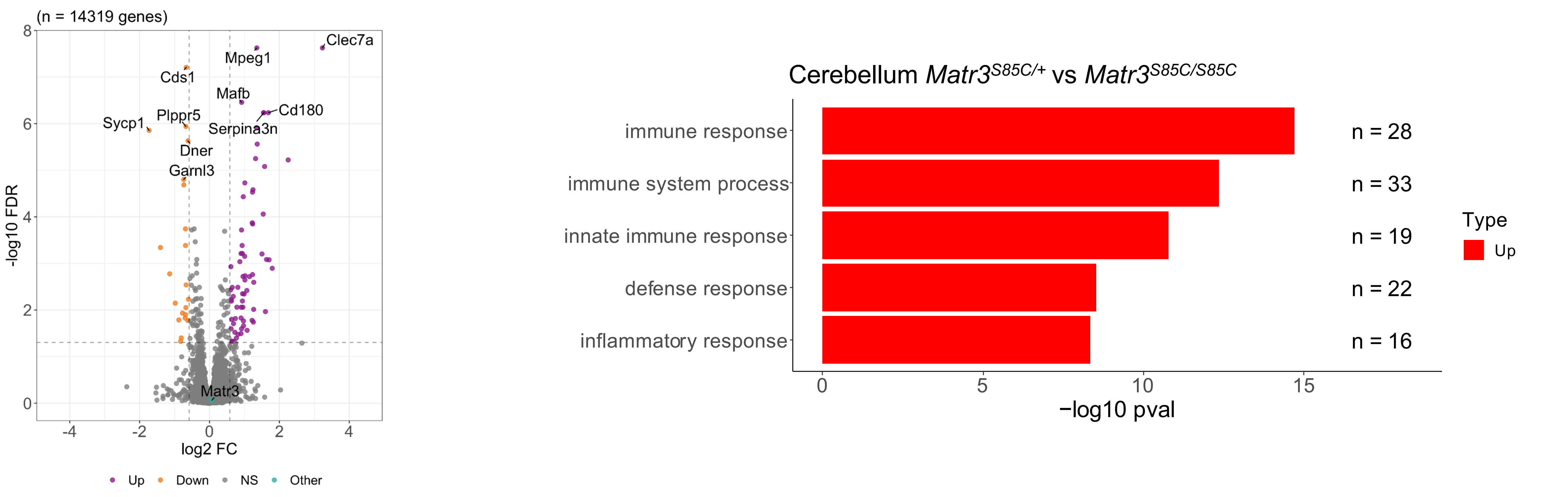

b Cerebellum *Matr3*$^{+/+}$ vs *Matr3*$^{S85C/+}$

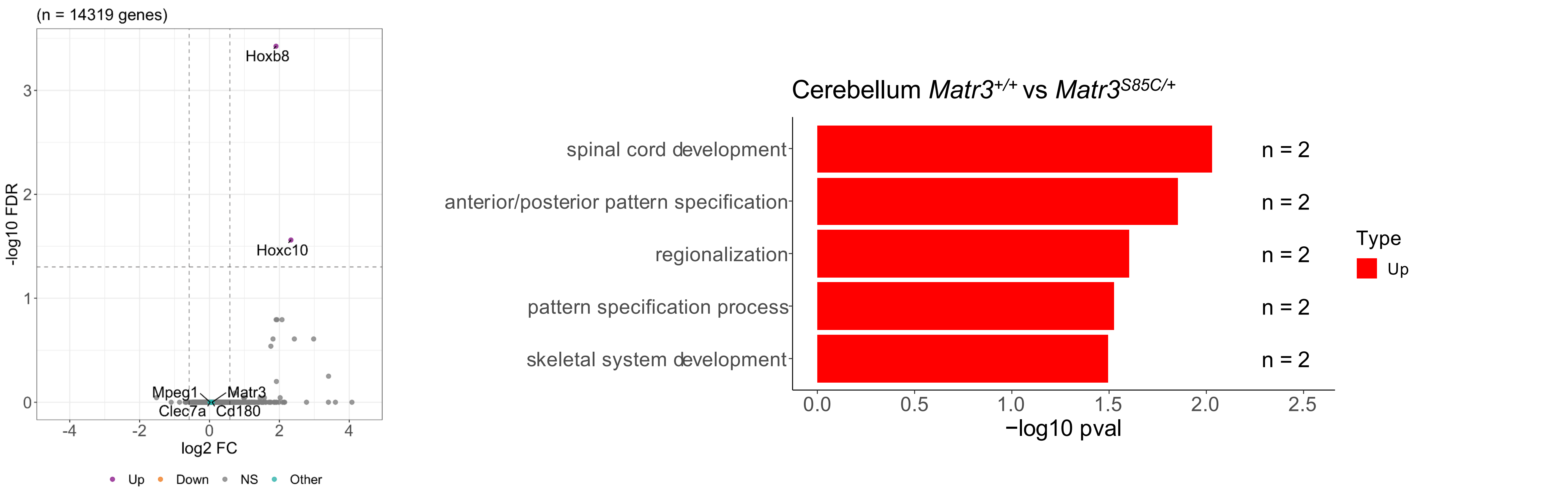

c Spinal cord *Matr3*$^{S85C/+}$ vs *Matr3*$^{S85C/S85C}$

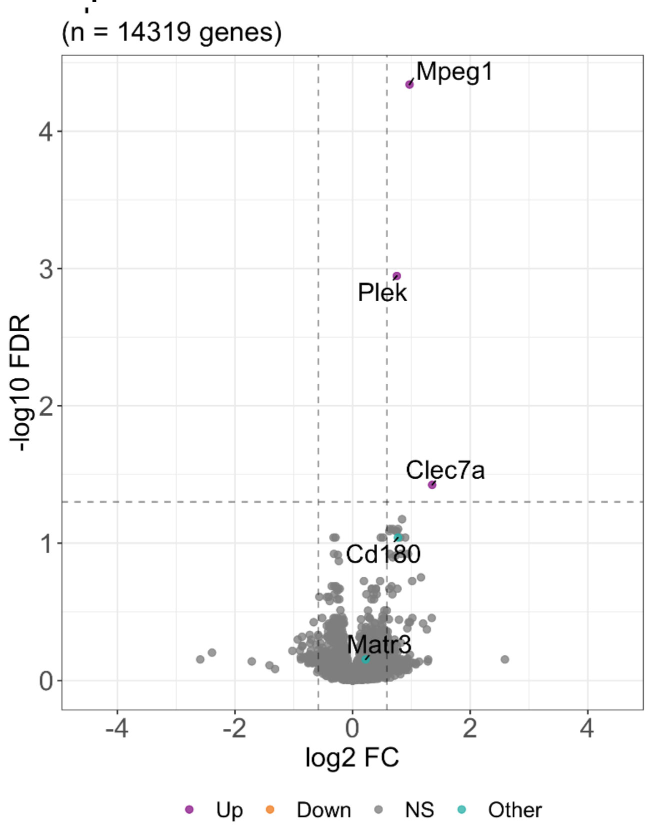

d Spinal cord *Matr3*$^{+/+}$ vs *Matr3*$^{S85C/+}$

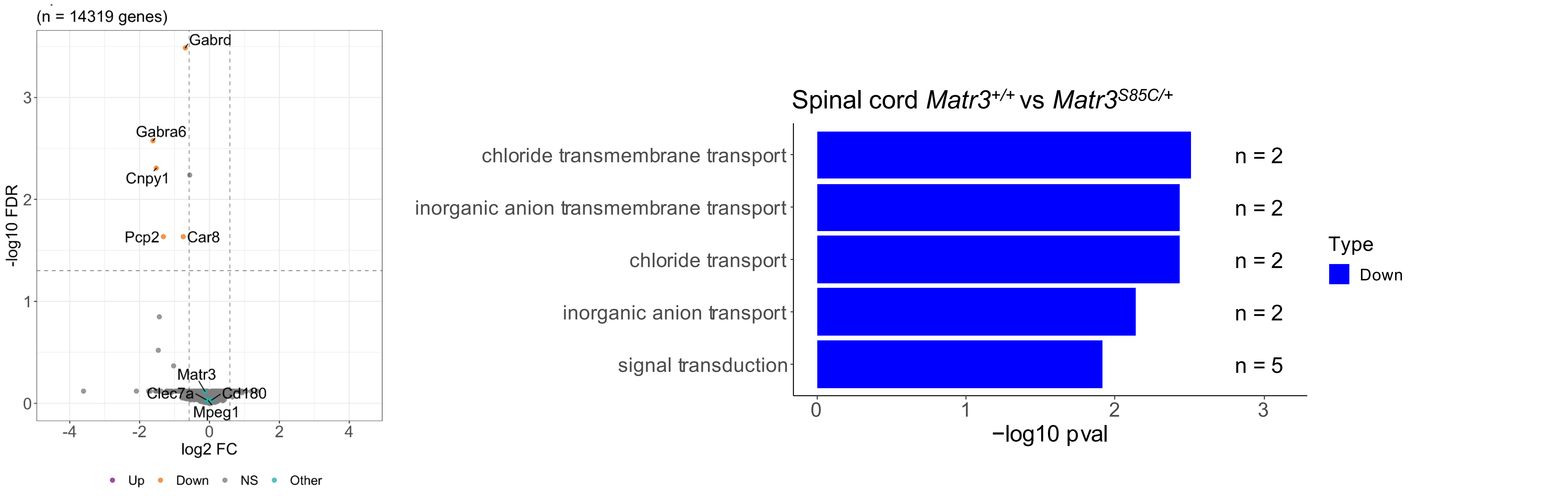

Supplementary Fig. 13 RNA-seq reveals differentially expressed genes in the cerebellum and spinal cord between the genotypes at 8-10 weeks old. **a** Volcano plot showing differentially expressed genes between heterozygous and homozygous S85C cerebellum. The five most upregulated or downregulated genes are noted with gene names. For the upregulated genes, GO analysis revealed enrichment of immune response-related processes. GO analysis found no enrichment for the downregulated genes. **b** Volcano plot showing differentially expressed genes between wildtype and heterozygous S85C cerebellum. The two most upregulated genes are indicated, which are related to spinal cord development. **c** Volcano plot showing differentially expressed genes from comparison between heterozygous and homozygous S85C lumbar spinal cord. The three most upregulated genes are indicated. **d** Volcano plot showing differentially expressed genes between wildtype and heterozygous S85C lumbar spinal cord. The five most downregulated genes are indicated. GO analysis for downregulated genes revealed enrichment in chloride transmembrane transport. Source data are provided as a Source data file.

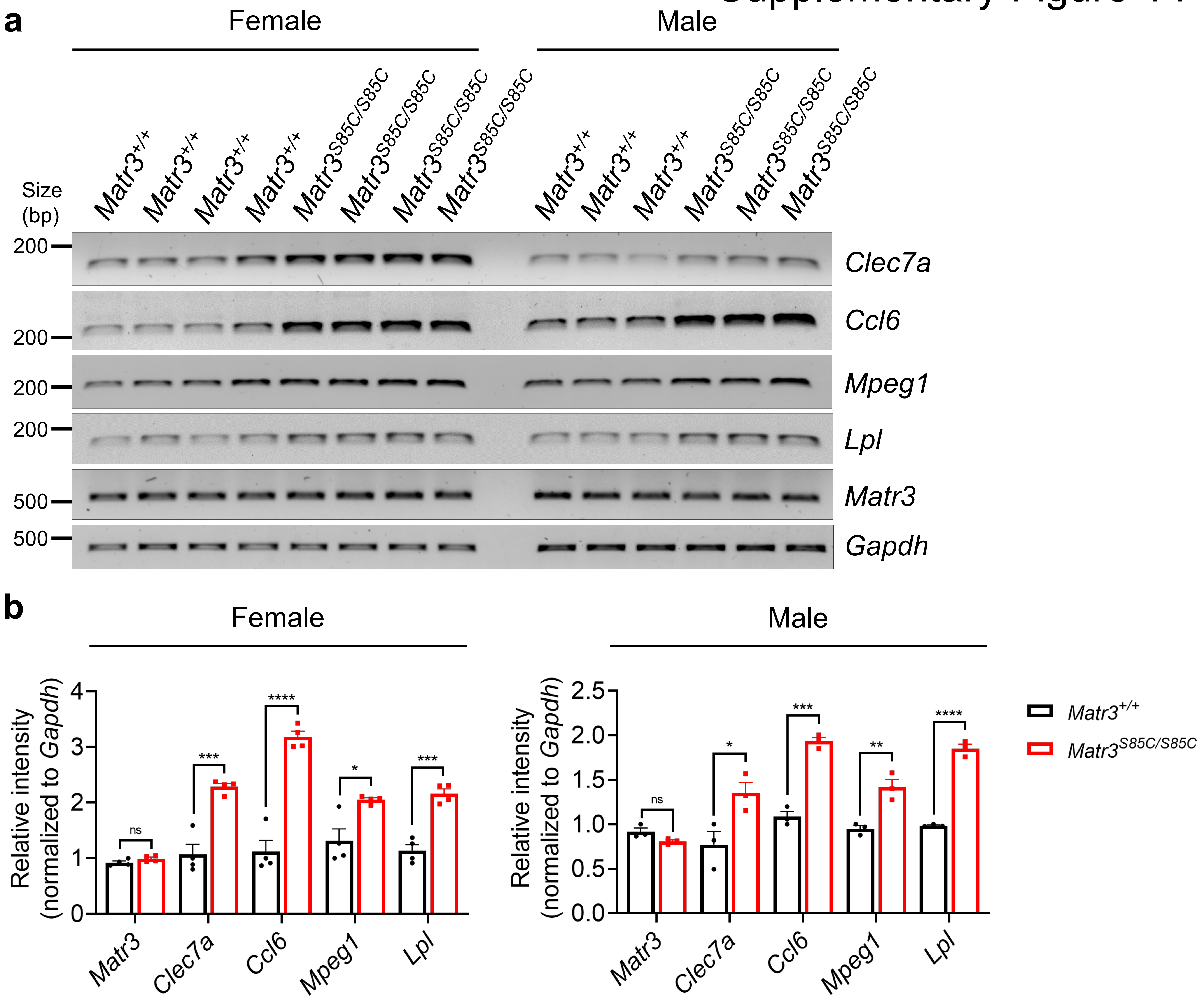

Supplementary Fig. 14 RT-PCR shows upregulation of immune response genes in the cerebellum of homozygous S85C mice at 8-10 weeks**. a** Representative images of RT-PCR results showing mRNA levels of immune response genes in the cerebellum at 8-10 weeks of age. *Matr3* and *Gapdh* primers were used as controls. **b** Quantification of RT-PCR results using Image J, normalized to *Gapdh*. Data is represented as mean ± s.e.m. with each dot representing a single animal (n=4 for females and n=3 for males). For females, *p*= not significant (ns), 0.0007 (***), <0.0001 (****), 0.0143 (*), 0.0003 (***) for *Matr3*, *Clec7a, Ccl6, Mpeg1* and *Lpl*, respectively (unpaired two-tailed *t*-test), and for males *p*= not significant (ns), 0.0389 (*), 0.0003 (***), 0.0078 (**), <0.0001 (****) for *Matr3*, *Clec7a, Ccl6, Mpeg1* and *Lpl* respectively (unpaired two-tailed *t*-test). Source data are provided as a Source data file.

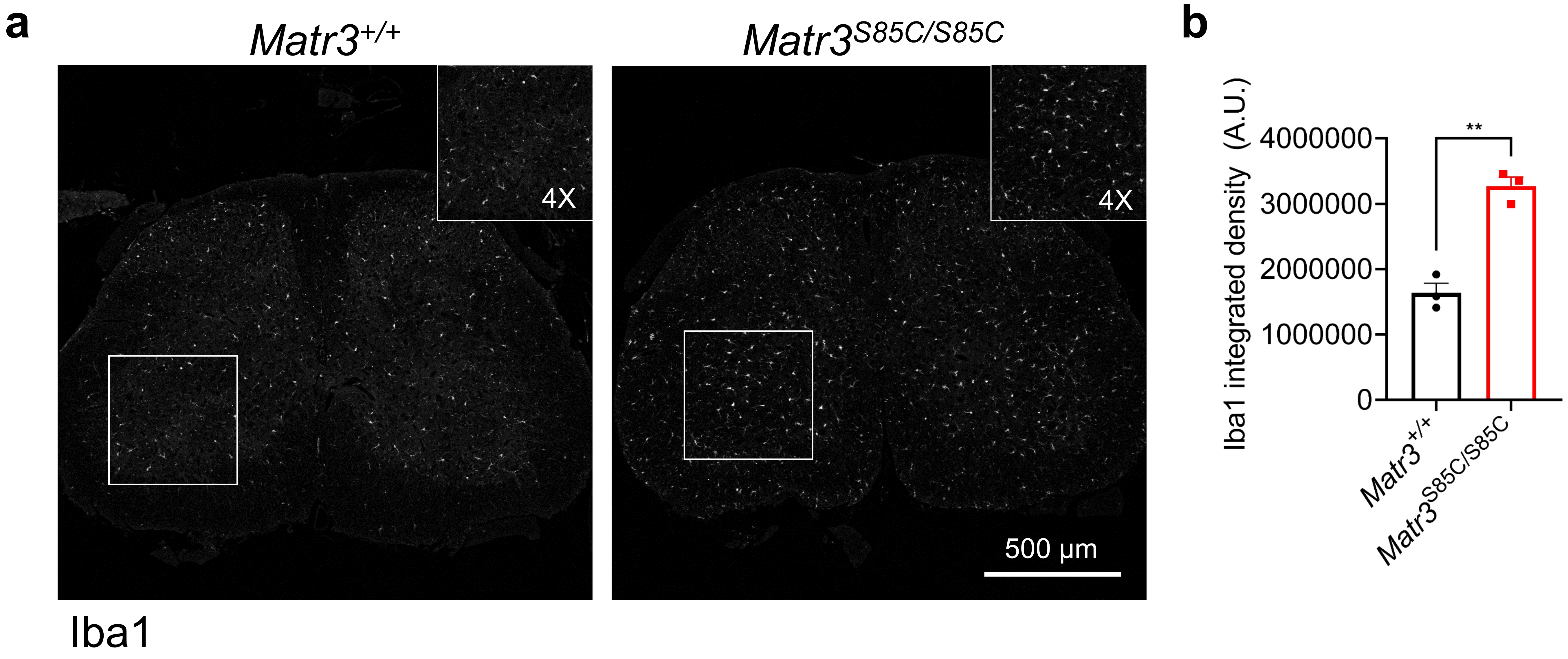

Supplementary Fig. 15 Lumbar spinal cord tissue from 30-week-old *Matr3*$^{S85C/S85C}$ mice show significantly increased Iba1-positive signals compared to wildtype littermates. **a** Representative images of Iba1 staining. Insets show 4X magnified images of boxed region of spinal cord. **b** Quantification of Iba1 intensity (n= 3 *Matr3*$^{+/+}$, 3 *Matr3*$^{S85C/S85C}$, ** p = 0.0013). Data shown as mean ± s.e.m., where each dot represents a single mouse. Significance was determined by unpaired two-tailed *t*-test. Source data are provided as a Source data file.

Supplementary Figure 16

a

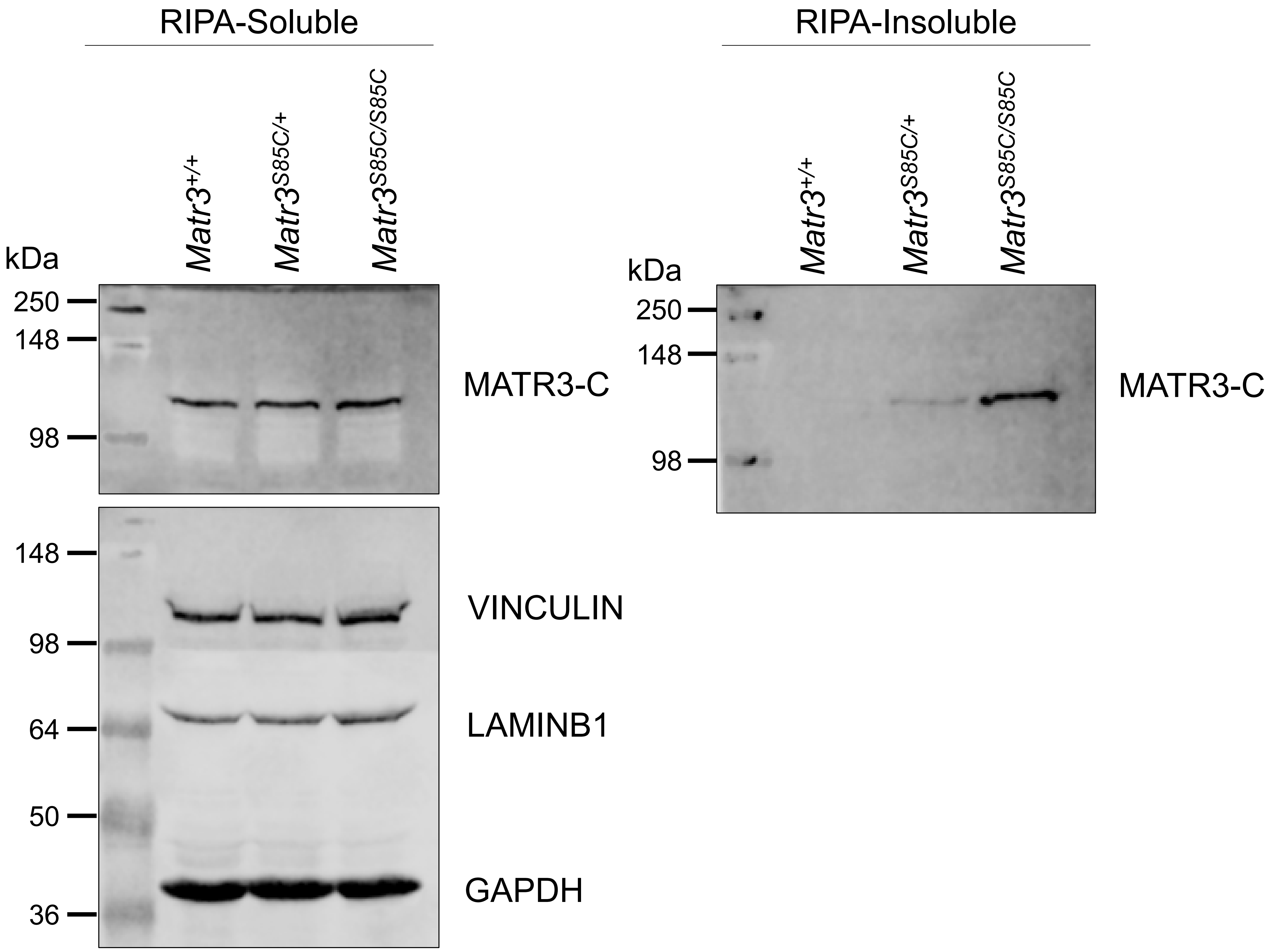

b

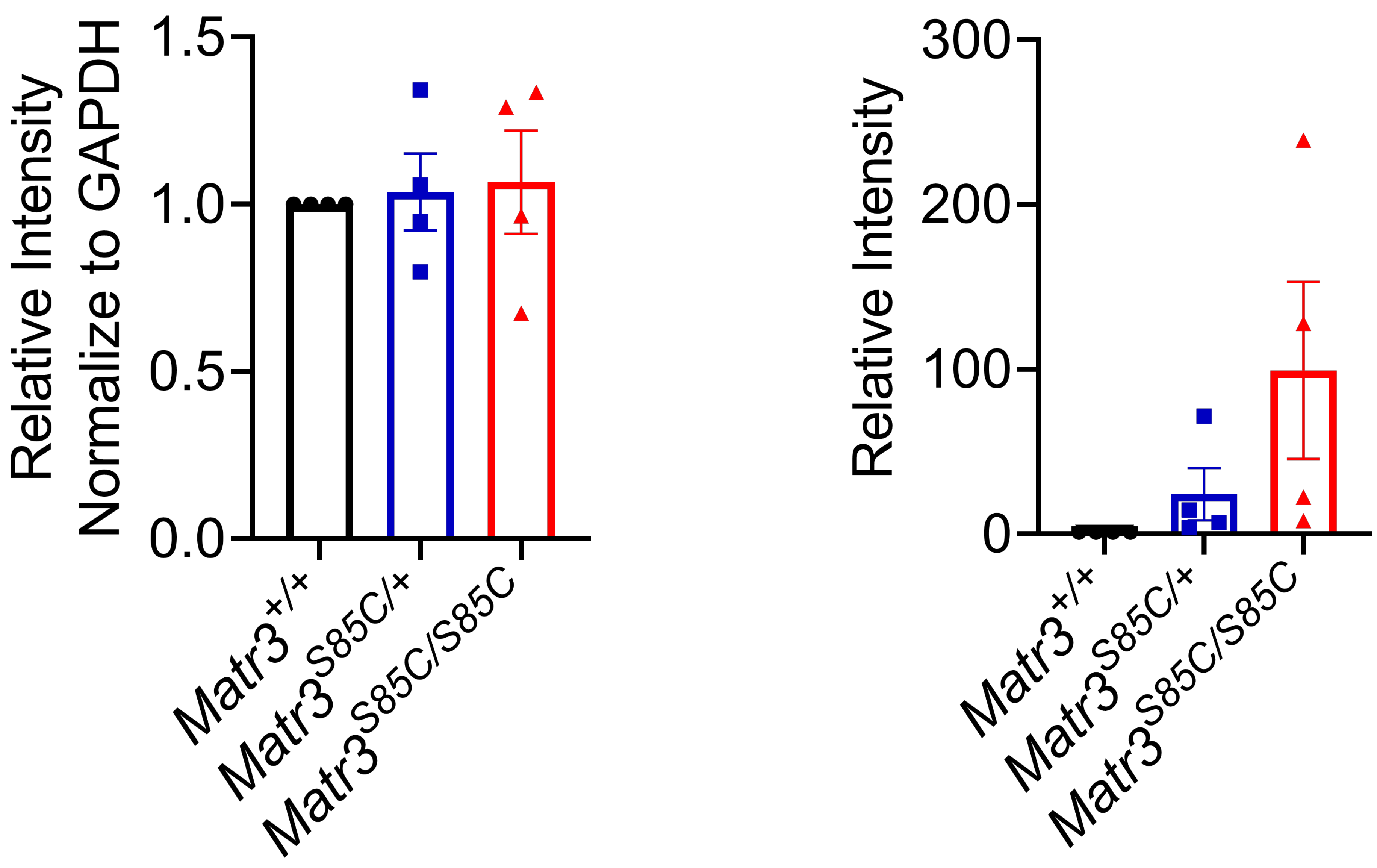

Supplementary Fig. 16 Solubility of MATR3 protein in the cerebellum of mice at disease end-stage. **a** Representative blot showing RIPA-soluble and RIPA-insoluble (urea-solubilized) MATR3 expression (detected with MATR3-C antibody). **b** Quantification of RIPA-soluble and RIPA-insoluble MATR3 using Image Studio Lite from four independent experiments. Data is shown as mean ± s.e.m. whereby each dot represents a single data point. Significance was determined using unpaired two-tailed *t*-test. Western blots revealed an increasing trend in MATR3 levels in the RIPA-insoluble fraction in *Matr3*$^{S85C/+}$ and *Matr3*$^{S85C/S85C}$ cerebellum compared to *Matr3*$^{+/+}$ cerebellum. This result supports the previous finding that the S85C mutation decreases MATR3 protein solubility (Malik *et al*. *eLife* 2018)[1.] However, the protein levels we observed through western blot analysis do not coincide with the results we observed through immunostaining where MATR3 levels are markedly decreased in Purkinje cells in *Matr3*$^{S85C/S85C}$ cerebellum (**Fig. 6a, c**). We think that this discrepancy is due to testing with bulk cerebellar tissue where majority of cells are granule cells in which MATR3 is not lost. Source data are provided as a Source data file.

Supplementary Table 1: List of primers used in this study

Gene	Forward Primer	Reverse Primer	Purpose
Intron of Ptprk	AAAACTGATCGGGACATTGC	GTGAAATCTGGAGCCAGGAG	Off-target sequencing confirmation
Rgs7	AACAATTTGACGGCCAAAAA	ACATTTGGCAGGTCTGAAGG	Off-target sequencing confirmation
Blcap	CAATGAGGAAGCTCCTGGTG	TGAATGTGTGTTGTGTGTGTT	Off-target sequencing confirmation
Erlin1	CATGGAGAAAATAGAACACACA CA	GAAGCGCCATTGTGAAAGAT	Off-target sequencing confirmation
Wapl	CCTTTTTCAGGCTCATTTGC	CATTGAATATGGTCATGGTCTCA	Off-target sequencing confirmation
Chromosome 13 Position 119138995	TACCAGGCTTCACAACCACA	AACGCAAAGGAACTTCAGGA	Off-target sequencing confirmation
Clhc1/1700034F02Rik	TGAAATACAAAAGGCAAACTAA TTCTT	CCTAATTTTACTTACTTTTCCTGG GTA	Off-target sequencing confirmation
Matr3 Exon 1	GCGTGCCGAACTAGAACTG	CCACTGCACTCCTAACATGG	Sequence validation
Matr3 Exon 2	TTGTTAAATTTCAGCATGAAAAG C	GAATTTGGGGCAAGTTCTCA	Sequence validation
Matr3 Exon 2	AGAGGTCCACTCCCTTTGTCT	TCCAGTGGATGACAGCTTTG	Sequence validation
Matr3 Exon 3	TGGTCCACAGCTGAGTGCT	AAATCCCCCAATCCAACAAT	Sequence validation
Matr3 Exon 4	TGGTGCCTGAAGTTCTTTTTG	CCGAGTGCTGGCAATAATTT	Sequence validation
Matr3 Exon 5	AAAGTACCAGTGTAAGGAATTT TCAGA	CCACCATTTACACTAAAGGCAGT	Sequence validation
Matr3 Exon 6	TCCATTTGTATTTGAAAGATGTC C	GGCAAAAGCAGTTGGATCTC	Sequence validation
Matr3 Exon 7	AGCAGGAATACCCACACACA	TTGACAATTAAAAATAACACCAAA A	Sequence validation
Matr3 Exon 8	TGAAAGGTTTGTGATTCCTAAG T	AGGCAAGGACAAATGAAAGG	Sequence validation
Matr3 Exon 9	CCCTGGTTTTGTTTTTGCAT	TGCTTGATCATGGGCATCTA	Sequence validation
Matr3 Exon 10	AGGGCATGGAATGAAACAAG	ACTGAGCCCATGATTTTTGC	Sequence validation
Matr3 Exon 11	AGGGAGGTTTGTGCTGTGTT	CGGCGTGACCAGTTTTATTA	Sequence validation
Matr3 Exon 12	ACTGCAGCTGACCTGGTCTT	TCTTGAACTGTACCCGAGATACC	Sequence validation

Matr3 Exon 13	TCAAGGCCAACCTGGTCTAC	CAGAAAAGGGGACGAGAAAA	Sequence validation
Matr3 Exon 14	GGCCATACTGGAATTCACACA	CCTTAAAGCCCAGGCAATTT	Sequence validation
Matr3 Exon 15	TGTAATTCTTTTTCCCCCTTCA	TGGTCAAAGAGAACGGGAAT	Sequence validation
Matr3 Exon 15	TGGAAAACAACTTGCACCAC	AAAAAGCCACAACGGACAAA	Sequence validation
Matr3 Exon 15	AATTCTTGACGCACGCTTCT	CTCCCAAGTGCTGGGAATAA	Sequence validation
Matr3	ACTGCAGCCTTGCTAGTTT	ATGCGAGGTCTCACCAAAAA	Genotyping MATR3 S85C mice with MseI restriction endonuclease digestion
Matr3	GCGTTACCATTTTTGAAGCAA	CCTCTACTTCCAATATTGAATATG C	Genotyping MATR3 S85C mice with simple allele-discriminating PCR (SAP) assay
Matr3	GGAGCTCATAGTGCACTGTCTT	TTGTCCTCTGGATAGCGACTC	Genotyping MATR3 KO mice with allelic discrimination PCR assay
Matr3	CTGGCCCCTTACAAGAGAGA	CACTGGCTTGCCAAACACTA	RT-PCR
Gapdh	ACTCCACTCACGGCAAATTC	CCTTCCACAATGCCAAAGTT	RT-PCR
Clec7a	CTTGCCTTCCTAATTGGATC	GCATTAATACGGTGAGACGATG	RT-PCR
Ccl2	AAGAAGATCGTCGCTATAACCC T	GCTTAGGCACCTCTGAACTCTC	RT-PCR
Mpeg1	CAGTCGTCTGGAATGTAAAAAG	GACTGTGCATTTGTCATAGGG	RT-PCR
Lpl	ATGGATGGACGGTAACGGGAA	CCCGATACAACCAGTCTACTACA	RT-PCR

Supplementary Table 2: Animal numbers (n) and significance values for Figure 2a

	Males					Females				
Age (weeks)	n Matr3$^{+/+}$	n Matr3$^{S85C/+}$	n Matr3$^{S85C/S85C}$	p-value	Sig.	n Matr3$^{+/+}$	n Matr3$^{S85C/+}$	n Matr3$^{S85C/S85C}$	p-value	Sig.
3	17	11	8	0.9993	ns	12	12	10	0.978	ns
5	17	11	8	0.7050	ns	12	12	10	0.9999	ns
7	17	11	8	0.5427	ns	12	12	10	0.9712	ns
9	17	11	8	0.2984	ns	12	12	10	0.875	ns
11	17	13	8	0.1551	ns	13	13	11	0.8181	ns
13	17	13	8	0.0644	ns	13	13	11	0.7623	ns
15	17	11	8	0.0182	*	12	12	10	0.5785	ns
17	17	11	8	0.0034	**	13	13	11	0.3902	ns
19	17	13	8	0.0002	***	11	13	11	0.1588	ns
21	17	13	8	<0.0001	****	10	13	11	0.1066	ns
23	17	13	8	<0.0001	****	13	12	11	0.0285	*
25	17	13	7	<0.0001	****	13	13	11	0.0086	**
27	17	13	7	<0.0001	****	13	13	11	0.0041	**
29	11	10	5	<0.0001	****	11	13	9	0.007	**
31	15	14	8	<0.0001	****	13	11	11	0.0009	***
33	14	12	7	<0.0001	****	9	13	11	0.0002	***
35	15	15	8	<0.0001	****	12	10	10	0.0003	***
37	11	13	7	<0.0001	****	11	12	8	0.0001	***
39	14	11	5	<0.0001	****	12	10	11	<0.0001	****
41	14	12	5	<0.0001	****	12	12	11	<0.0001	****
43	14	13	7	<0.0001	****	12	12	11	<0.0001	****
45	11	14	7	<0.0001	****	12	12	11	<0.0001	****
47	14	14	7	<0.0001	****	10	12	11	<0.0001	****
49	14	14	7	<0.0001	****	10	12	11	<0.0001	****
51	11	14	7	<0.0001	****	10	12	11	<0.0001	****
53	11	14	7	<0.0001	****	10	12	11	<0.0001	****
55	10	13	7	<0.0001	****	10	12	11	<0.0001	****
57	10	13	6	<0.0001	****	10	12	11	<0.0001	****
59	10	13	6	<0.0001	****	10	12	11	<0.0001	****
61	10	13	6	<0.0001	****	8	10	9	<0.0001	****

Supplementary References

1. Malik AM, Miguez RA, Li X, Ho YS, Feldman EL, Barmada SJ. Matrin 3-dependent neurotoxicity is modified by nucleic acid binding and nucleocytoplasmic localization. *Elife* **7**, (2018).
