# Supplementary material for: Selective neuronal degeneration in MATR3 S85C knock-in mouse model of early-stage ALS
Source: Nat Commun. 2020 Oct 20;11:5304. doi: 10.1038/s41467-020-18949-w (PMC7576598; doi:10.1038/s41467-020-18949-w)
Supplement: Supplementary file 3 — Description of Additional Supplementary Files [file 41467_2020_18949_MOESM3_ESM.pdf]

Description of Additional Supplementary Files

File Name: Supplementary Data 1
Description: Predicted off-targets from S85C gRNA. Off-targets were predicted using an algorithm which takes the effect of gRNA:target DNA mismatches into account. Mutations expected to occur in a gene or predicted to have only two mismatches were PCR amplified (with indicated primers) and sequence verified.

File Name: Supplementary Data 2
Description: Sequence validation of all Matr3 exons. Primers were designed within the intron regions between the 15 exons of mouse Matr3 in order to PCR amplify and verify the sequences of the splice sites and exons.

File Name: Supplementary Data 3
Description: Weekly Animal Monitoring Score Sheet. Body condition (BCS), weight changes (BW), mobility and activity (M/A), appearance/hydration (A/H), respiratory problem (R) and severe ataxia (A) were measured weekly to biweekly and scored as indicated. Actions required are indicated for each score range.

File Name: Supplementary Data 4
Description: MATR3 antibodies used for immunohistochemistry and summary of results.

File Name: Supplementary Data 5
Description: Upregulated and downregulated gene lists from RNA-seq data.

File Name: Supplementary Movie 1
Description: Homozygous MATR3 S85C knock-in mouse shows severe motor defects.

File Name: Supplementary Movie 2
Description: Homozygous MATR3 S85C knock-in mouse shows hindlimb dragging phenotype.
