# Supplementary material for: Selective neuronal degeneration in MATR3 S85C knock-in mouse model of early-stage ALS
Source: Nat Commun. 2020 Oct 20;11:5304. doi: 10.1038/s41467-020-18949-w (PMC7576598; doi:10.1038/s41467-020-18949-w)
Supplement: Supplementary file 11 — Reporting Summary [file 41467_2020_18949_MOESM11_ESM.pdf]

nature research

Corresponding author(s): Jeehye Park

Last updated by author(s): 8/26/2020

Reporting Summary

Nature Research wishes to improve the reproducibility of the work that we publish. This form provides structure for consistency and transparency in reporting. For further information on Nature Research policies, see Authors & Referees and the Editorial Policy Checklist .

Statistics

For all statistical analyses, confirm that the following items are present in the figure legend, table legend, main text, or Methods section.

n/a	Confirmed	
☐	☒	The exact sample size (*n*) for each experimental group/condition, given as a discrete number and unit of measurement
☐	☒	A statement on whether measurements were taken from distinct samples or whether the same sample was measured repeatedly
☐	☒	The statistical test(s) used AND whether they are one- or two-sided<br>*Only common tests should be described solely by name; describe more complex techniques in the Methods section.*
☒	☐	A description of all covariates tested
☐	☒	A description of any assumptions or corrections, such as tests of normality and adjustment for multiple comparisons
☐	☒	A full description of the statistical parameters including central tendency (e.g. means) or other basic estimates (e.g. regression coefficient) AND variation (e.g. standard deviation) or associated estimates of uncertainty (e.g. confidence intervals)
☐	☒	For null hypothesis testing, the test statistic (e.g. *F*, *t*, *r*) with confidence intervals, effect sizes, degrees of freedom and *P* value noted<br>*Give P values as exact values whenever suitable.*
☒	☐	For Bayesian analysis, information on the choice of priors and Markov chain Monte Carlo settings
☒	☐	For hierarchical and complex designs, identification of the appropriate level for tests and full reporting of outcomes
☒	☐	Estimates of effect sizes (e.g. Cohen's *d*, Pearson's *r*), indicating how they were calculated

Our web collection on statistics for biologists contains articles on many of the points above.

Software and code

Policy information about availability of computer code

Data collection	No software was used for data collection.
Data analysis	GraphPad Prism (version 7.00)<br>ImageStudio Lite (v 5.2)<br>ImageJ (v1.52p)<br>Fiji with ImageJ (v 1.52n)<br>FastQC (v 0.11.7)<br>trimmomatic (v 0.39)<br>STAR aligner (v 2.6.0c)<br>QualiMap (v 2.2.1)<br>featureCounts (v 1.6.3)<br>R (v.3.6.0)<br>edgeR (v 3.26.5)<br>ggfortify (v 0.4.9)<br>gProfileR (v 0.6.7)

For manuscripts utilizing custom algorithms or software that are central to the research but not yet described in published literature, software must be made available to editors/reviewers. We strongly encourage code deposition in a community repository (e.g. GitHub). See the Nature Research guidelines for submitting code & software for further information.

Data

Policy information about availability of data

All manuscripts must include a data availability statement. This statement should provide the following information, where applicable:

- Accession codes, unique identifiers, or web links for publicly available datasets
- A list of figures that have associated raw data
- A description of any restrictions on data availability

Authors can confirm that all relevant data are included in the paper and its supplementary information. Source data are provided with this paper. The source data underlying Figs 1d-f, 2a-b, 3a-i, 4b-c, 4e-f, 5b, 5d, 5f, 5h, 6c-d, 6f-g, 6i and 7g-h, and Supplementary Figs 1c, 2a, 6a-c, 7a-c, 7e-g, 8b, 12c-f, 14a-b, 15b and 16a-b are provided as a Source Data file. RNA seq data has been deposited in the ArrayExpress database https://identifiers.org/arrayexpress:E-MTAB-8838. The raw immunofluorescence and immunohistochemistry images generated and/or analyzed during the current study are available in the figshare repository https://doi.org/10.6084/m9.figshare.12824312. All other relevant data are available from the authors upon request.

Field-specific reporting

Please select the one below that is the best fit for your research. If you are not sure, read the appropriate sections before making your selection.

☒ Life sciences ☐ Behavioural & social sciences ☐ Ecological, evolutionary & environmental sciences

For a reference copy of the document with all sections, see nature.com/documents/nr-reporting-summary-flat.pdf

Life sciences study design

All studies must disclose on these points even when the disclosure is negative.

Sample size	Sample sizes are consistent with those that have been generally employed in previous studies with ALS models; e.g. PMID: 31591561; PMID: 31056746<br>The sample size employed was sufficient to achieve the most biologically significant change.
Data exclusions	No data was excluded from the study.
Replication	Each experiment was performed at least 3 times and reported in the manuscript. All attempts at replication were successful.
Randomization	Mice for behavioral tests, brain and spinal cord histology, RT-PCR, Western blot analysis, RNA seq were randomly chosen from each genotype.
Blinding	Authors performing mouse experiments including behavioral tests, histology, RT-PCR, Western blot, RNA seq was blinded to group allocation. Quantification for the immunohistology data (Figures 4-6) was performed in a blinded manner.

Reporting for specific materials, systems and methods

We require information from authors about some types of materials, experimental systems and methods used in many studies. Here, indicate whether each material, system or method listed is relevant to your study. If you are not sure if a list item applies to your research, read the appropriate section before selecting a response.

Materials & experimental systems

n/a	Involved in the study	
☐	☒	Antibodies
☒	☐	Eukaryotic cell lines
☒	☐	Palaeontology
☐	☒	Animals and other organisms
☒	☐	Human research participants
☒	☐	Clinical data

Methods

n/a	Involved in the study	
☒	☐	ChIP-seq
☒	☐	Flow cytometry
☒	☐	MRI-based neuroimaging

Antibodies

Antibodies used	Rabbit polyclonal anti-MATR3 antibody, C-terminal (Abcam, ab84422)<br>Rabbit polyclonal anti-MATR3 antibody, N-terminal (Sigma, HPA036565)<br>Mouse monoclonal anti-MATR3 antibody, clone 2539C3a, (SantaCruz, sc81318)<br>Goat polyclonal anti-ChAT antibody (Millipore-Sigma, ab144P)<br>Rabbit polyclonal anti-lamin B1 antibody (Proteintech, 12987-1-AP)

Mouse monoclonal anti-NeuN antibody, clone A60 (Millipore-Sigma, mab377)
Mouse monoclonal anti-calbindin D28K antibody, clone CB300 (SWant, 300)
Rabbit polyclonal anti-myelin PLP antibody (Abcam, ab28486)
Mouse monoclonal anti-neurofilament antibody, clone SMI-312 (BioLegend, 837904)
Rabbit polyclonal anti-synaptophysin I antibody (Synaptic Systems, 101 002)
Rabbit polyclonal anti-synapsin I antibody (Abcam, ab64581)
Chicken polyclonal anti-neurofilament H antibody (Abcam, ab4680)
Rabbit polyclonal anti-Iba1 antibody (Wako, 01919741)
Rabbit polyclonal anti-GFAP antibody (Abcam, ab7260)
Mouse monoclonal anti-GAPDH antibody, clone 6C5 (Millipore-Sigma, CB1001)

488 Alexa Fluor donkey anti-mouse (Thermofisher, A-21202)
488 Alexa Fluor donkey anti-rabbit (Thermofisher, A-21206)
488 Alexa Fluor goat anti-chicken (Thermofisher, A-11039)
555 Alexa Fluor donkey anti-mouse (Thermofisher, A-31570)
555 Alexa Fluor donkey anti-rabbit (Thermofisher, A-31572)
647 Alexa Fluor donkey anti-mouse (Thermofisher, A-31571)
647 Alexa Fluor donkey anti-goat (Thermofisher, A-21447)
Biotinylated goat anti-rabbit antibody (Vector Laboratories, BA-1000)
Goat anti-mouse IRDye 800CW (LiCor, 926-32210)
Goat anti-rabbit IRDye 800CW (LiCor, 926-32211)

Validation

All MATR3 antibodies used in this study (ab84422, HPA036565 and sc81318) were validated through western blotting and immunohistochemistry with MATR3 knockout mice (Supplementary Figure 2 in our manuscript).

Rabbit anti-MATR3 C-terminal ab84422) was previously validated for IHC-P and ICC/IF from (manufacturer's website) and for WB and ICC/IF (human) in Raigor D et al. Identification of novel nesprin-1 binding partners and cytoplasmic matrin-3 in processing bodies. Mol Biol Cell 27:3894-3902 (2016).

Rabbit anti-MATR3 N-term (HAP036565) was previously validated for IHC, IF and WB using the Human Protain Atlas (HPA) project (from the manufacturer's website), and was used for WB and IHC (human) in Johnson JD et al Mutations in the Matrin 3 gene cause familial amyotrophic lateral sclerosis. Nat neurosci 17:664-666 (2014), and for WB, IP and ICC (mouse and human) in Boehringer A et al ALS associated mutations in Matrin 3 alter protein-protein interactions and impede RNA nuclear export. Sci Rep 7:14529 (2017).

Mouse anti-MATR3 internal (sc81318) was previously tested for WB and IP (from the manufacturer's website), and was used for WB (mouse) in Fujita T and Fuijii H. Direct identification of insulator components by insertional chromatin immunoprecipitation. PLoS ONE 6:e26109 (2011), and WB, IHC, ICC/IF (human) in Tada M et al. Matrin 3 is a component of neuronal cytoplasmic inclusions of motor neurons in sporadic amyotrophic lateral sclerosis. Am J Pathol 188:507-514 (2018).

Goat anti-ChAT (ab144P) was previously tested for IHC, ICC and WB (from the manufacturer's website), and was used for IF/ICC (human) in Adams KL et al. Foxp1-mediated programming of limb-innervating motor neurons from mouse and human embryonic stem cells. Nat commun 14:6778 (2015), and IF (mouse) in Fayzullina S and Martin LJ. Skeletal muscle DNA damage precedes spinal motor neuron DNA damage in a mouse model of spinal muscular atrophy (SMA). PloS one 9:e93329 (2014).

Rabbit anti-Lamin B1 (12987-1-AP) was previously tested for ChIP, FC, ICC, IF, IHC-P, IP and WB (from the manufacturer's website), and was used for IHC (mouse) in Dong L et al. The E3 ubiquitin ligase c-Cbl inhibits microglia-mediated CNS inflammation by regulating PI3K/Akt/NF-kB pathway. CNS neurosci ther 22:661-669 (2016)., and IF (human) in Gasset-Rosa F et al. Polyglutamine-expanded Huntingtin exacerbates age-related disruption of nuclear integrity and nucleocytoplasmic transport. Neuron 94:48-57 (2017).

Mouse anti-NeuN (mab377) was previously tested for FC, ICC, IF, IHC, IP and WB (from the manufacturer's website), and was used for IHC (mouse) in Krzisch M et al. Pre-existing astrocytes form functional perisynaptic processes on neurons generated in the adult hippocampus. Brain struct funct 220:2027-2042 (2015) and Radford H et al. PERK inhibition prevents tau-mediated neurodegeneration in a mouse model of frontotemporal dementia. Acta neuropathol 130:633-642 (2015).

Mouse anti-Calbindin D28K (300) was previously tested for WB, IHC and ICC (from the manufacturer's website), and was used for IHC (mouse) in Agoglia A et al. Corticotropin-releasing factor receptor-1 neurons in the lateral amygdala display selective sensitivity to acute and chronic ethanol exposure. Eneuro 7:eneuro.0420-19.2020 (2020) and Prolidase enzyme is required for extracellular matrix integrity and impacts on postnatal cerebellar cortex development. J comp neurol 528:61-80 (2020).

Rabbit anti-myelin PLP (ab28486) was previously tested for IHC-P (from the manufacturer's website), and was used for IHC (mouse) in Tognatta R et al. Astrocytes are required for oligodendrocyte survival and maintenance of myelin compaction and integrity. Front cell neurosci 14:74 (2020) and Farhangi S et al. In vivo conversion of astrocytes to oligodendrocyte lineage cells in adult mice demyelinated brains by Sox2. Mult scler relat disord 28:263-272 (2019).

Mouse anti-Neurofilament (837904) was previously tested for IHC-P and WB (from the manufacturer's website), and was used for IHC (mouse) in Choi Y et al. Tuberous sclerosis complex proteins control axon formation. Genes dev 22:2485-2495 (2008) and Schulz A et al. Neuron-specific deletion of the Nf2 tumor suppresor impairs functional nerve regeneration. PLoS one 11:e0159718 (2016).

Rabbit anti-Synpatophysin I (101002) was previously tested for WB, IP, ICC, IHC, EM and ELISA (from the manufacturer's website), and was used for IHC (mouse) in Baczyk M et al. Synaptic restoration by cAMP/PKA drives activity-dependent neuroprotection to motoneurons in ALS. J exp med 2178:e20191734 (2020) and Wang S et al. Therapeutic potential of a TrkB agonistic antibody for Alzheimer's disease. Theranostics 1015:6854-6874 (2020).

Rabbit anti-Synapsin I (ab64581) was previously tested for WB, IHC-P and IP (from the manufacturer's website), and was used for IHC (mouse) in Jay TR et al. TREM2 is required for microglial instruction of astrocytic synaptic engulfment in neurodevelopment. Glia 67:1873-1892 (2019) and Stoffel W et al. SMPD3 deficiency perturbs neuronal proteostasis and causes progressive cognitive impairment. Cell death dis 9:507 (2018).

Chicken anti-Neurofilament H (ab4680) was previously tested for IHC and WB (from the manufacturer's website), and was used for IHC (mouse) in Chen B et al. Analysis of Schwann cell migration and axon regeneration following nerve injury in the sciatic nerve bridge. Front mol neurosci 12:308 (2019) and IHC (human) in Laug D et al. Nuclear factor I-A regulates diverse reactive astrocyte responses after CNS injury. J clin invest 129:4408-4418 (2019).
Rabbit anti-Iba1 (Wako-1919741) was previously tested for IHC and ICC/IF (from the manufacturer's website), was used for IHC (mouse) in Ziehn MO et al. Therapeutic testosterone administration preserves excitatory synaptic transmission in the hippocampus during autoimmune demyelinating disease. J neurosci 32:12312-12324 (2012) and ICC/IF (mouse, rat) in Hosmane S et al. Toll/interleukin-1 receptor domain-containing adapter inducing interferon-B mediates microglial phagocytosis of degenerating axons. J neurosci 32:7745-7757 (2012).
Rabbit anti-GRAP (ab7260) was previously tested for WB, IHC-P and ICC (from the manufacturer's website), was used for IHC (mouse) in Mehla J et al. Age-dependent behavioral and biochemical characterization of single APP knock-in mouse (APPNL-G-F/NL-G-F) model of Alzheimer's disease. Neurobiol aging 75:25-37 (2019) and WB (mouse) in Silverman JM et al. CNS-derived extracellular vesicles from superoxide dsmutase 1 (SOD1)G93A ALS mice orginate from astrocytes and neurons and carry misfolded SOD1. J biol chem 294:3744-3759 (2019).
Mouse anti-GAPDH (CB1001) was previously tested for ELSA, WB and IHC (from the manufacturer's website), was used for WB (human) in Gagarin D et al. Genomic profiling of acquired resistance to apoptosis in cells derived from human atherosclerotic lesions: potential role of STATs, cyclinD1, BAD, and Bcl-XL. J Mol Cell Cardiol 39:453-465 (2005) and Yang Z et al. Resistance to Fas-induced apoptosis in cells from human atherosclerotic lesions: elevated Bcl-XL inhibits apoptosis and caspase activation. J vasc res 44:483-494 (2007).

Animals and other organisms

Policy information about studies involving animals; ARRIVE guidelines recommended for reporting animal research

Laboratory animals

All mouse procedures were performed under the approval of the Animal Care Committee (ACC), and housed at The Centre for Phenogenomics (TCP), accredited by the Association for Assessment and Accreditation of Laboratory Animal Care International. Mice were kept on a 12 hour light/12 hour dark cycle, at 20-22°C and 43% humidity, with ad libitum access to food and water.

All mice have C57BL/6J background.

MATR3 S85C knock-in mice
Body weight: males and females, 3 - 61 weeks of age
Behavioral tests: males and females at 6, 10, 20, 30, 40, 50, 60 weeks or at end-stage
Immunohistochemistry: male/female at 6, 11-14, 30, 60 weeks or at end-stage
RNA seq: females at 8-10 weeks
RT-PCR and western: male/female at 3 weeks, 8-10 weeks or at end-stage

MATR3 knockout mice
Western blot and immunohistochemistry, embryos were used.

Wild animals

No wild animals were used.

Field-collected samples

The study does not involve samples collected from the field.

Ethics oversight

All mouse procedures were performed under the approval of the Animal Care Committee (ACC), and housed at The Centre for Phenogenomics (TCP), accredited by the Association for Assessment and Accreditation of Laboratory Animal Care International.

Note that full information on the approval of the study protocol must also be provided in the manuscript.
